# Uncovering Hidden Layers of Cell Cycle Regulation through Integrative Multi-omic Analysis

**DOI:** 10.1371/journal.pgen.1005554

**Published:** 2015-10-06

**Authors:** Ranen Aviner, Anjana Shenoy, Orna Elroy-Stein, Tamar Geiger

**Affiliations:** 1 Department of Cell Research and Immunology, George S. Wise Faculty of Life Sciences, Tel Aviv University, Tel Aviv, Israel; 2 Department of Human Molecular Genetics and Biochemistry, Sackler Faculty of Medicine, Tel Aviv University, Tel Aviv, Israel; Stanford University School of Medicine, UNITED STATES

## Abstract

Studying the complex relationship between transcription, translation and protein degradation is essential to our understanding of biological processes in health and disease. The limited correlations observed between mRNA and protein abundance suggest pervasive regulation of post-transcriptional steps and support the importance of profiling mRNA levels in parallel to protein synthesis and degradation rates. In this work, we applied an integrative multi-omic approach to study gene expression along the mammalian cell cycle through side-by-side analysis of mRNA, translation and protein levels. Our analysis sheds new light on the significant contribution of both protein synthesis and degradation to the variance in protein expression. Furthermore, we find that translation regulation plays an important role at S-phase, while progression through mitosis is predominantly controlled by changes in either mRNA levels or protein stability. Specific molecular functions are found to be co-regulated and share similar patterns of mRNA, translation and protein expression along the cell cycle. Notably, these include genes and entire pathways not previously implicated in cell cycle progression, demonstrating the potential of this approach to identify novel regulatory mechanisms beyond those revealed by traditional expression profiling. Through this three-level analysis, we characterize different mechanisms of gene expression, discover new cycling gene products and highlight the importance and utility of combining datasets generated using different techniques that monitor distinct steps of gene expression.

## Author Summary

How the genetic program of a cell unfolds to execute complex functions depends on a dynamic interplay between multiple steps that include transcription of DNA into mRNA, translation of mRNA into protein and post-translational degradation of mature proteins. Profiling of gene expression is traditionally based on measurements of steady-state mRNA levels, but recent studies have shown that mRNA and protein levels are highly discordant, suggesting that post-transcriptional mechanisms play a dominant role in modulating protein abundance. Here we combine measurements of mRNA, translation and protein across the mammalian cell cycle to uncover the hidden complexity of cell cycle regulation. Using this approach, we gain insights into the dynamics of protein synthesis and degradation and identify new genes and functions that cycle through cell division by periodic changes in translation or degradation rates. Integrative multi-omic analyses combining information on the transcriptome, translatome and proteome hold great promise for providing transformative biological insights in a variety of model systems.

## Introduction

The flow of genetic information from DNA to protein entails multiple regulated steps that include mRNA transcription, processing and export, followed by translation into proteins that undergo folding, post-translational modification and eventually degradation. Steady-state protein abundance therefore reflects a dynamic net outcome of this long series of processes. Microarray and RNA sequencing (RNA-seq) measurements of mRNA levels are often used to profile gene expression as a proxy of function, assuming that protein and mRNA expression are highly concordant. However, advances in proteomic techniques that allow system-wide comparison of proteomes and transcriptomes have revealed that the relationship between mRNA and protein levels is much more complex than previously expected, resulting in limited correlations (reviewed in [1]). This is thought to reflect the widespread effects of post-transcriptional mechanisms e.g. translational control and protein degradation. Therefore, a multi-level analysis that explores not only steady-state mRNA and protein levels but also translation and degradation rates can shed new light on the complex nature of gene expression.

Several methods exist for measuring translation at a system wide level. Most recently, we introduced a mass-spectrometric (MS) method called PUromycin-associated Nascent CHain Proteomics (PUNCH-P) [2], which quantifies the amount of nascent polypeptide chains associated with translating ribosomes. This allows direct measurement of translation products with high temporal resolution, without the potentially confounding effects of lengthy in-vivo labeling using modified amino acids, as required for techniques such as pulsed stable isotope labeling in cell culture (pSILAC) [3], quantitative non-canonical amino acid tagging (QuaNCAT) [4] and stochastic orthogonal recoding of translation with chemoselective modification (SORT-M) [5]. Alternatively, translation can also be monitored by RNA-seq of ribosome-associated mRNAs or ribosome-protected mRNA fragments using methods such as TRAP [6] or ribosome profiling [7], which generate a prediction of newly-synthesized proteins based on mRNA templates.

In recent years, the relationship between transcription, translation and protein degradation has been investigated in mammalian models such as LPS-stimulated dendritic cells, resting fibroblasts, differentiating monocytes and lymphoblastoid cell lines [8–11], and several other studies have generated matching mRNA/protein time series [12–14] (for a recent review of published multi-omic datasets see [15]). Here we report a combined analysis of gene expression along the mammalian cell cycle, which requires tight regulation to ensure accurate and well-coordinated DNA replication and cell division. It has been shown that specific phases of the cell cycle involve wide-spread dynamic reprogramming of gene expression at multiple discrete levels, including transcription and mRNA degradation [16], translation [17,18], post-translational modification [19,20] and protein degradation, primarily through the ubiquitin-proteasome pathway [21,22]. Regardless of whether mRNA levels, translation or protein abundance were monitored, most of these studies found that approximately 5–10% of measured gene products oscillate across the cell cycle. However, no publication has provided a detailed account of the complex interplay between mRNA, translation and protein levels. In this work we combined microarray, PUNCH-P and total proteome measurements in a HeLa cell cycle model to characterize the dynamic relationship between the transcriptome, translatome and proteome, uncovering complex regulatory patterns.

## Results

### Comparison of mRNA, translation and protein levels along the cell cycle supports a significant role for post-transcriptional control

Aiming to better understand how transcription, translation and protein degradation change along the cell cycle, we combined triplicate measurements of the transcriptome (Affymetrix microarray [23]), translatome (PUNCH-P [2]) and proteome (MS; this study) from synchronized HeLa S3 cells. For all three measurements, cells were synchronized to G1, S and G2/M phases of the cell cycle using double thymidine block, which induces an arrest at the G1/S checkpoint and thus establishes a common baseline for synchronous progression [24]. Synchronized populations were then harvested at specific time points without further addition of drugs or agents that can affect gene expression and confound comparisons between the different phases [24] (for analysis of synchronization efficiency, see S1 Fig). mRNA and protein were experimentally normalized by analyzing the same amounts of biological material at each phase of the cell cycle. Translation measurements were normalized by the amount of translating ribosomes at each phase and therefore reflect relative changes in synthesis of specific proteins, rather than the absolute fluctuations in protein synthesis that occurs as cells progress through mitosis [25,26]. To allow direct comparison of absolute abundance between the different types of gene products, we used robust multi-array average (RMA) for mRNA levels and intensity-based absolute quantification (iBAQ) for both translatome and protein abundance. The combined dataset of mRNA, translation and protein levels consisted of nearly 7,000 gene products that were detected in the transcriptome as well as the translatome and/or proteome (S1 Table).

First, to confirm that relative protein stability can be inferred from our data without directly measuring degradation rates, we used published protein half-life measurements from cycling HeLa cells [27] and generated a color-coded scatterplot, in which each protein is colored based on its previously-reported stability (S2 Fig). Proteins with long half-lives were indeed found to cluster predominantly above the regression line, indicating steady-state accumulation, whereas proteins with short half-lives were found mostly below the line, indicating rapid degradation. 1D functional enrichment analysis [28] based on relative protein stability score, i.e. the ratio of steady-state abundance to translation level of each protein, found that relatively stable proteins were enriched for processes e.g. glycolysis, amino acid biosynthesis and oxidation-reduction, while labile proteins were enriched for transcription, membrane association, cell adhesion and regulation of cell cycle (S2 Table). These findings are consistent with previous estimates generated using pSILAC [9].

**Fig 1:**
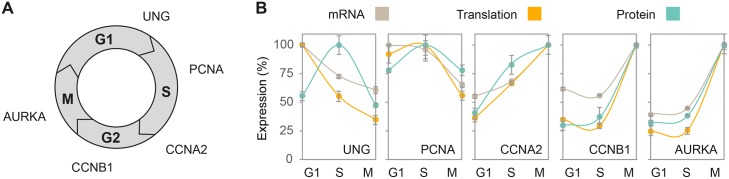
Concordant expression patterns of cell cycle markers. (**A**) Schematic representation of the order in which known cell cycle markers peak at the protein level. (**B**) Line plots represent the mean change in mRNA (gray), translation (orange) and protein (blue) levels for cell cycle markers from (A) at G1, S and G2/M phases, with peak abundance set as 100%. G1, S and G2/M phases correspond to timepoints 2, 8 and 12h for mRNA and 2, 8.5 and 14h for translation and protein. Error bars represent +SD of triplicate measurements.

Next, we examined whether typical cell cycle markers share a common pattern of mRNA, translation and protein expression. To that end, we selected genes known to peak sequentially from G1 to mitosis at the mRNA and/or protein level. These include Uracil-DNA glycosylase (UNG), which peaks prior to the G1/S checkpoint [29]; Proliferating cell nuclear antigen (PCNA) and Cyclin A2 (CCNA2), which peaks in early and late S-phase, respectively [30,31]; Cyclin B1 (CCNB1), which peaks in G2/M [32]; and Aurora kinase A (AURKA), which peaks during mitosis [33] (Fig 1A). For each marker, we plotted the proportion of its mRNA, translation and protein measurements at G1, S and G2/M, with peak level defined as 100%. Similar patterns were observed at the three levels of expression for all markers (Fig 1B), except for UNG, whose mRNA and translation levels peaked during G1 while protein abundance peaked at S-phase. This difference in pattern may reflect a lag time between accumulation of mRNA and its encoded protein, which was observed for many gene products [13,21,34]. It may also be attributed to the rapid proteasomal degradation of UNG outside of S-phase, a mechanism thought to prevent accumulation of UNG protein prior to the initiation of DNA replication [35]. To further validate that the published microarrays reflect the same cell synchrony as the samples used for the translatome and proteome analysis, we generated mRNA measurements using real-time PCR (qPCR) and compared them to the microarray profiles. Analysis of the same cell cycle markers showed comparable expression patterns between the published microarray data and our qPCR results (S3 Fig), confirming similar cell cycle periodicity despite the independent measurements.

**Fig 2:**
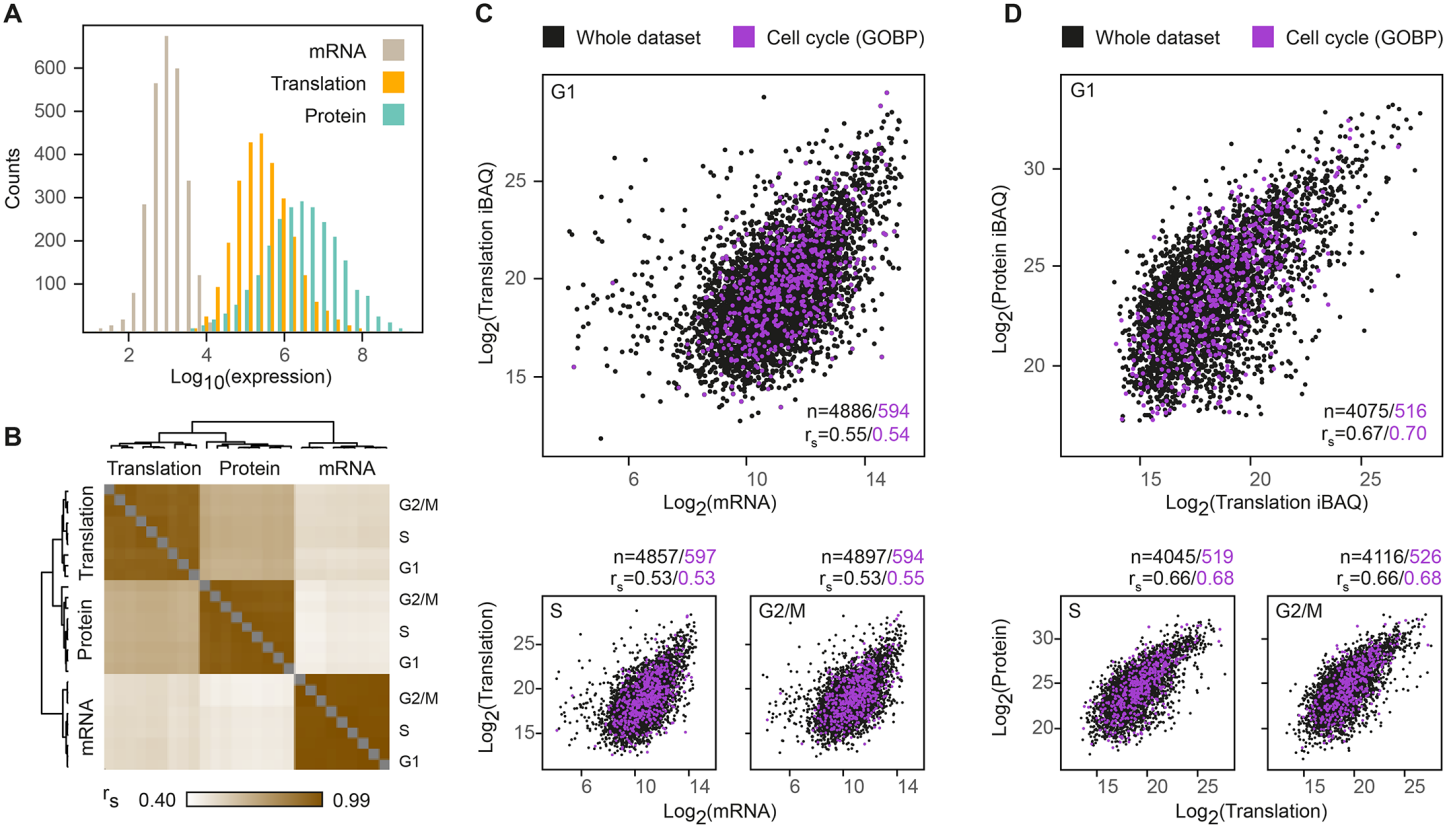
Widespread post-transcriptional control across the cell cycle. (**A**) Histogram of RMA-normalized mRNA (gray), iBAQ-normalized translation (orange) and TOP3-normalized protein (blue) levels throughout the cell cycle. (**B**) Hierarchical clustering of Spearman’s rank correlations for mRNA, translation and protein levels at G1, S and G2/M phases of the cell cycle. (**C-D**) Scatterplots of mean logarithmized mRNA versus translation levels (C), and translation versus protein levels (D) for G1 (top), S and G2/M (bottom). Gene products with GOBP cell cycle annotations are highlighted purple.

As multiple copies of protein are produced from a single mRNA molecule, we expected translation products to have a larger dynamic range as compared to mRNAs. Indeed, translation products spanned almost 5 orders of magnitude, compared to less than 4 for mRNA and 6 for steady-state protein levels (Fig 2A), suggesting that regulation of both translation and protein degradation contributes to the diversity in protein abundance. Since the dynamic range of microarrays is lower than that of RNA-seq [36], this comparison may underestimate the true dynamic range of the transcriptome. However, the translatome and steady-state proteome were both generated using similar MS conditions and the differences in their range are therefore presumed to be biologically relevant and not merely a technical artifact. Next, we calculated individual Spearman’s rank correlations for mRNA, translation and protein levels at each cell cycle phase. Hierarchical clustering of the correlation coefficients showed that each level of expression best correlated with itself across the three phases, confirming that the bulk of gene products remains relatively invariant along the cell cycle (Fig 2B). mRNA levels and protein abundance show significant but limited positive correlation (r_s_ = 0.46–0.48) throughout the cell cycle, consistent with previously reported values for similar comparisons in mammalian cells [9,21,37,38]. The correlations between translation and protein levels were higher (r_s_ = 0.66–0.67, Fig 2B) throughout the cell cycle, reflecting the combined contribution of translation rates and mRNA levels. To verify that the higher correlation between the translatome and the proteome is not overestimated due to the technical similarities between measurement techniques, i.e. MS-based analysis and use of iBAQ to determine absolute protein abundance, we employed an alternative absolute quantification method based on the summed intensities of the top 3 peptides per protein (TOP3 [39]). This was not found to have a significant impact on the correlations (S4 Fig), suggesting that the high translatome-proteome correlation is not merely due to the use of a similar quantification method. Even if the true correlation is lower, this comparison indicates that post-translational processes e.g. protein degradation have a considerable contribution to the variance in steady-state protein levels. Furthermore, to control for the technical variability that is inherent to each measurement platform, we followed the procedure described by Csardi et al [40] to generate corrected Spearman’s correlations. With our data, because the intra-platform correlations were high (r_s_ = 0.95–0.99), this correction did not have a significant effect (S5 Fig). Overall, these results suggest that mRNA, translation and protein degradation all play a significant role in modulating steady-state protein abundances.

To determine whether known cell cycle genes share distinct distribution patterns, we filtered our data for cell cycle functions based on Gene Ontology Biological Process (GOBP). Overall, gene products with cell cycle annotations had a similar distribution and only marginally higher correlations as compared to the entire dataset, at all phases (Fig 2C and 2D). While this may indicate that cell cycle gene products do not share common modes of regulation at the level of translation or protein degradation, it may also reflect confounding inter-platform effects resulting from the different measurement techniques. Our downstream analyses aimed to minimize these effects.

### Fold-change ratios of mRNA, translation and protein levels show cell cycle-specific patterns

**Fig 3:**
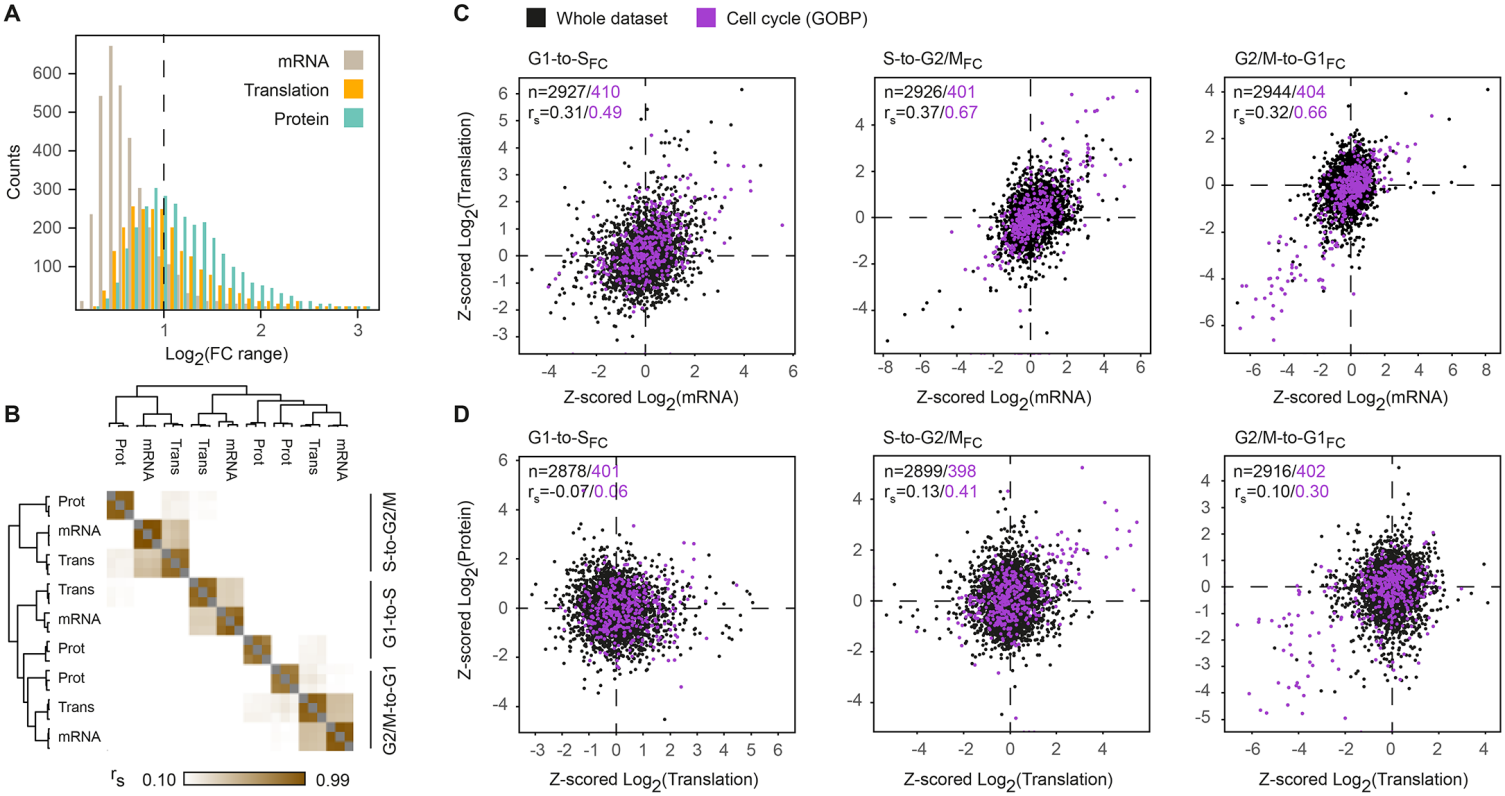
Fold-change analysis of mRNA, translation and protein levels along the cell cycle. (**A**) Histogram of the range of fold-change ratios for RMA-normalized mRNA (gray), LFQ-normalized translation (orange) and LFQ-normalized protein (blue) levels throughout the cell cycle. (**B**) Hierarchical clustering of Spearman’s rank correlations of the Z-scored fold-change values between cell cycle phases, for mRNA, translation and protein levels. Each pair of consecutive cell cycle phases is labeled G1-to-S_FC_, S-to-G2/M_FC_ and G2/M-to-G1_FC_. (**C-D**) Scatterplots of mean Z-transformed fold-change ratios of mRNA versus translation levels (**C**) and translation versus protein levels **(D)**. Gene products with GOBP cell cycle annotations are highlighted purple.

To minimize potential biases related to platform-specific effects or differences in dynamic range between mRNA, translation and protein measurements, we compared the change in gene expression along the cell cycle within each platform. We calculated fold-change (FC) ratios of mRNA, translation and protein levels for each pair of consecutive cell cycle phases, reflecting the transition from G1 to S-phase, S-phase to mitosis, and mitosis to G1 (labeled G1-to-S_FC_, S-to-G2/M_FC_ and G2/M-to-G1_FC_, respectively). Similar to the range of absolute expression measurements, the range of fold-change ratios across the cell cycle was also higher for translation compared to mRNA and for protein compared to translation (Fig 3A), suggesting that regulation of translation and protein stability contributes not only to the diversity of steady-state protein abundance but also to the magnitude of changes upon transition between phases. This analysis of fold-changes eliminates much of the inter-platform variance, but is still influenced by the dynamic range of the measurement technique, which was shown to be relatively low for microarrays. To ensure that mRNA-level regulation is not underestimated, we performed Z-score transformation of each sample, unifying the dynamic range of all fold changes. However, while this normalization aims to reduce artifacts associated with the experimental methodology, it assumes that the true biological range of mRNA and proteins is identical and therefore carries an inherent risk of overestimating the contribution of mRNA while underestimating the impact of translational regulation and protein degradation.

In contrast to absolute measurements that best correlated within each level of expression, Z-transformed fold-change ratios reveal higher similarity within each cell cycle phase and between the different levels of expression. Highest correlations were observed between mRNA and translation fold-changes, particularly as cells progress in and out of mitosis (Fig 3B, S-to-G2/M_FC_ and G2/M-to-G1_FC_). While changes in mRNA levels were associated with concordant changes in their translation, such changes were not readily reflected in protein abundance, as evidenced by the surprisingly low correlations between translation and protein fold-changes (Fig 3B). In this dynamic system of the cell cycle, it is possible that changes in steady-state protein abundance lag behind mRNA translation and therefore escape detection.

Compared to the entire dataset, GOBP cell cycle genes showed considerably higher correlations in all pairwise comparisons, with maximum correlations of r_s_ = 0.67 and r_s_ = 0.66 for mRNA and translation fold-change ratios upon entry to and exit from mitosis, respectively. In other words, the expression of most genes with annotated cell cycle functions changes concordantly at both the mRNA and translation levels, primarily during mitosis, without notable differences in translation efficiency of individual mRNAs. These gene products seem to be highly specific to mitosis, as the same subset of genes is first up-regulated when cells progress into mitosis and then down-regulated upon transition to G1 (S6 Fig). This group consists of many gene products with known mitotic functions e.g. cyclins, mitotic kinases, kinesins, spindle assembly and chromosome segregation proteins (S3 Table).

### Clustering of fold-change ratios reveals complex interplay between mRNA, translation and protein levels along the cell cycle

**Fig 4:**
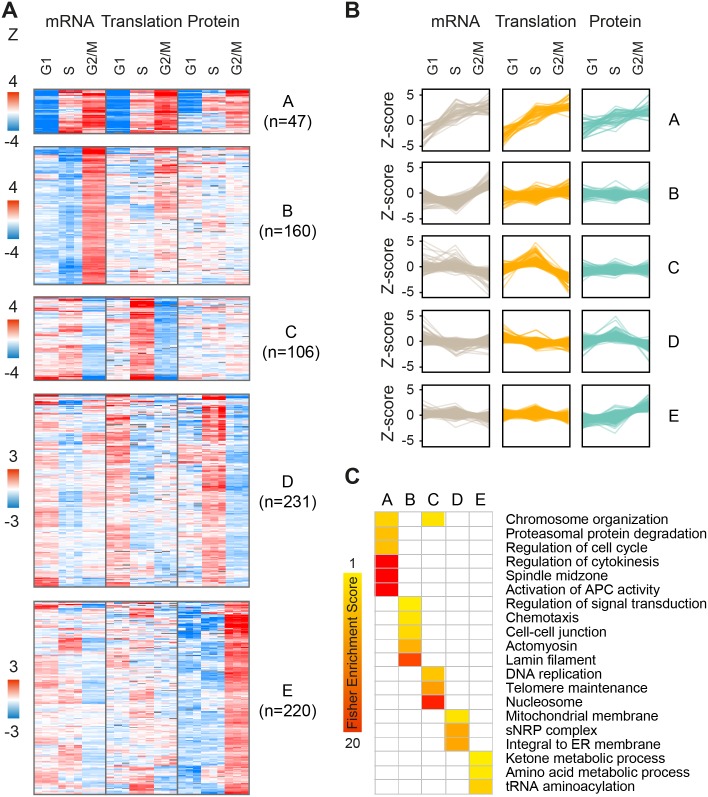
Clustering of periodic gene products. (**A-B**) K-means clustering of gene products showing statistically-significant (one-sample T-test of fold-changes, FDR<0.05) changes along the cell cycle in at least one of mRNA, translation and/or protein levels. Each panel represents a distinct cluster with a separate heatmap (**A**) and profile plot (**B**) reporting Z-transformed values for fold-change mRNA, translation and protein levels. G1, S and G2/M represent fold-change ratios relative to the previous cell cycle phase i.e. G2/M-to-G1, G1-to-S, and S-to-G2/M, respectively. (**C**) Fisher enrichment scores for the clusters A-E (FDR <0.02, selected categories). The complete enrichment analysis is included in S5 Table.

As cell cycle patterns were readily detected in the fold-change but not absolute expression datasets, we next sought to explore the interplay between changes in mRNA levels, translation and protein abundance for all gene products that fluctuate along the cell cycle, regardless of prior cell cycle annotation. For this purpose, we filtered our dataset for gene products that showed statistically significant changes (one-sample T-test of Z-transformed fold-changes, FDR<0.05) in any of the cell-cycle phases, at the level of mRNA, translation or protein or any combination thereof. Filtration of the data resulted in a subset of n = 2,323 significantly changing proteins (S4 Table). We then performed k-means clustering of gene products into ten clusters with distinct patterns of expression. The first five clusters showed the highest fold-changes (Fig 4, clusters A-E), while the other five showed more subtle differences (S7 Fig, clusters F-J). These clusters reveal three main modes of interplay between the regulatory levels: concordant, lagging or one-level cycling of the transcriptome, translatome or proteome.

For example, while clusters A and E both show peak protein expression during mitosis, this results from different modes of regulation. Cluster A is characterized by concordant increases in mRNA, translation and protein during mitosis (47 genes; Fig 4A and 4B); it is enriched for annotations such as cell division, cytokinesis, spindle and microtubule-based movement (Fig 4C), and consists of genes with known mitotic functions, including cyclins and kinases, mitotic checkpoint (MCC) proteins, kinesins and ubiquitin modifiers (e.g. CCNB1 and CCNB2, PLK1, BUBR1, CDC20, KIF11 and UBE2C). On the other hand, cluster E (220 genes; Fig 4A and 4B) consists of proteins that peak during mitosis and then drop back down upon transition to either G1 or S-phases, but these fluctuate almost entirely at the steady-state protein level, suggesting post-translational control of protein stability. This cluster is not enriched for classical mitotic processes, but rather for tRNA synthetases and metabolic functions e.g. carbohydrate and amino acid metabolism (e.g. PGK1, IDH1, MDH1, PHGDH, PFKM, TALDO1). Interestingly, genes included in cluster C (106 genes; Fig 4A and 4B) oscillate primarily at the level of translation, peaking upon transition to S-phase and declining upon entry to mitosis. Some of these genes also show mild transcript fluctuations, peaking at either G1 or S-phase. This cluster is enriched for S-phase related functions e.g. telomere maintenance, chromosome organization and DNA replication (e.g. RFC4, PARP2, POLA1, FANCI). In contrast, cluster D (231 genes; Fig 4A and 4B) includes gene products such as the known S-phase marker Geminin (GMNN), whose mRNA and translation levels increase at G1 while their steady-state protein abundance lags behind and peaks at S-phase. This cluster is enriched for mitochondria and endoplasmic reticulum related processes (for the complete list of enriched terms, see S5 Table).

To confirm that the cycling patterns observed here were not artificially introduced by the Z-score normalization, we performed a similar analysis using the non-normalized fold-change data. We filtered for gene products with a minimum 1.5 fold-change in at least 2 out of 3 measurements of mRNA, translation or protein, to emphasize the effect of fold-changes rather than statistical significance. Unsupervised hierarchical clustering of these filtered fold-change ratios (n = 832, S6 Table) revealed two main clusters of peak S-phase or peak mitosis expression, with cycling patterns strikingly similar to those generated by the clustering of Z-scored data (S8 Fig). However, one pattern was significantly more prevalent in this type of analysis; about 40% of gene products (n = 339) showed increased protein accumulation during mitosis, compared to only 10% (n = 220) in the Z-scored dataset (cluster E). Considering that the dynamic range of the translatome and proteome is not biased due to inter-platform differences (both are MS-based), this analysis further supports a robust role for regulation of post-translational protein stability along the cell cycle.

### Effect of cell growth on quantification of gene expression

**Fig 5:**
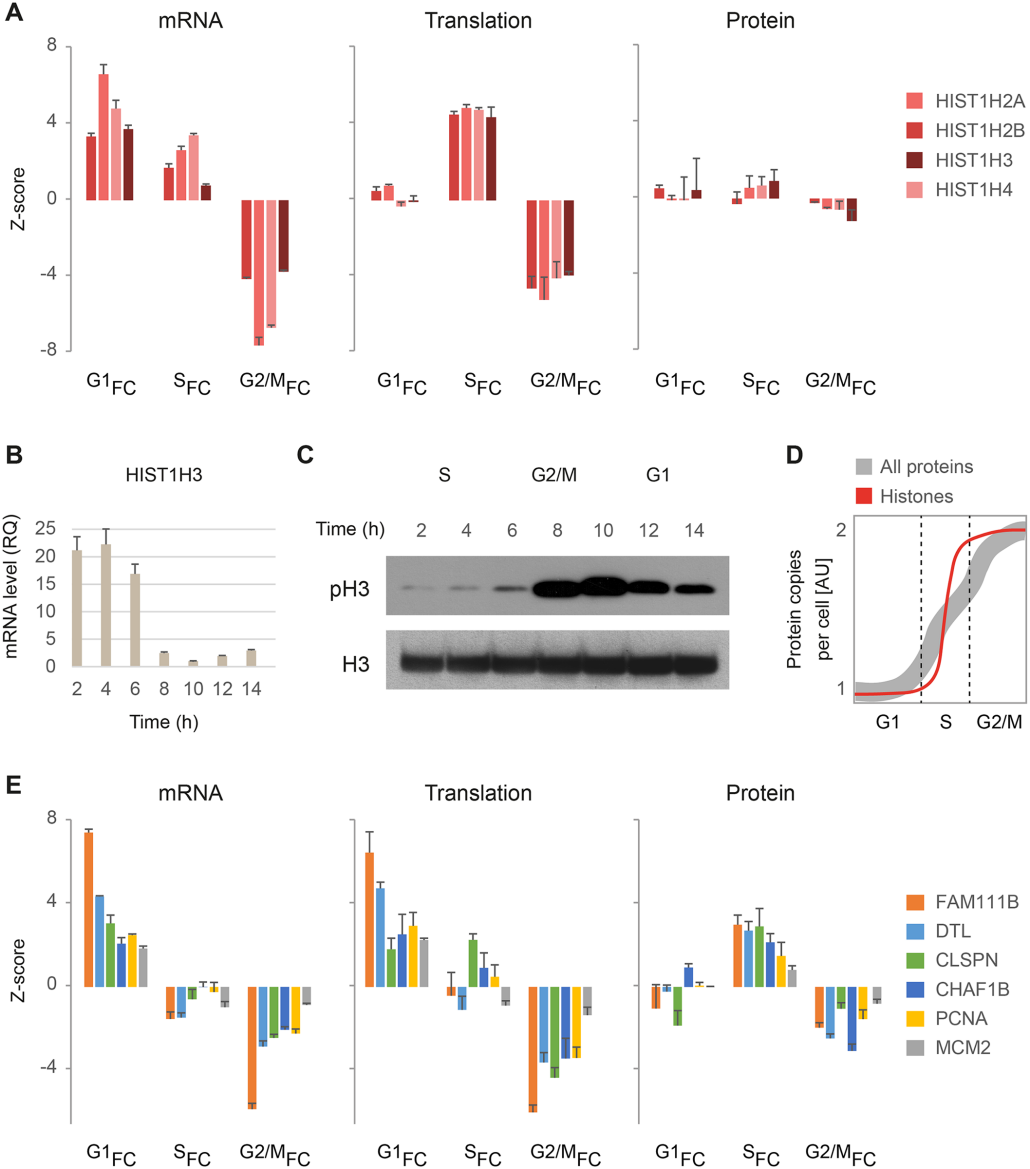
Analysis of S-phase regulation. (**A**) mRNA, translation and protein fold-change ratios for core histones along the cell cycle. G1_FC_, S_FC_ and G2/M_FC_ represent fold-change ratios relative to the previous cell cycle phase i.e. G2/M-to-G1, G1-to-S, and S-to-G2/M, respectively. Bars represent means +SD of triplicate measurements. (**B-C**) HeLa cells were synchronized using double-thymidine block and harvested at 2, 4, 6, 8, 10, 12 and 14 hours after release from the second block. RNA and protein were extracted and subjected to qPCR (**B**) and immunoblot (**C**) analysis using primers and antibodies specific to Histone H3. (**D**) Schematic representation of growth rates along the cell cycle, reflected as increase in protein copies per cell. The gray line represents an average growth rate for the entire proteome in HeLa cells, as measured experimentally (adapted from [43]). The red line represents hypothetical proteins that function during DNA replication and must therefore be produced specifically at S-phase. (**E**) mRNA, translation and protein fold-change ratios for gene products showing peak G1 expression at the mRNA and translation levels, followed by peak protein accumulation at S-phase.

Next, we took a closer look at the pattern of expression in cluster C. The gene products in this cluster show a considerable boost in translation levels during S-phase, but the increase in protein synthesis is not reflected by steady-state protein accumulation. Histones, a major gene group in this cluster, are known to be produced during DNA replication to coat the newly-synthesized chromatin. Histone expression is tightly regulated at multiple levels, including transcription, mRNA stability and translation, to ensure accurate duplication and prevent accumulation of excess unincorporated histones that can be toxic for the cells (reviewed in [41]). While the mRNA levels of all core histones (H2A, H2B, H3 and H4) drastically increased at G1, their translation levels lagged behind and increased only at S-phase (Fig 5A). This is not surprising, because translation of histone mRNAs requires the stem-loop binding protein (SLBP), which is only present at S-phase (our data and [42]). Nevertheless, while histone mRNA and translation levels fluctuate dramatically along the cell cycle, steady-state protein levels remain relatively static (Fig 5A). We validated these findings by experimentally measuring mRNA and protein dynamics of histone H3 in synchronized cells. Although qPCR showed that H3 mRNA accumulates during S-phase and is subsequently degraded (Fig 5B), immunoblot analysis showed that its protein levels remain stable as cells progress from S-phase to mitosis, as evidenced by the increase in H3 phosphorylation (Fig 5C). While increased protein degradation can explain this pattern, we hypothesize that given the known roles of histones in S-phase, the increase in protein synthesis may be obscured by dilution effects related to cell growth. As cells progress from G1 to mitosis, the content of the cytoplasm is doubled in preparation for cell division, with growth rates increasing exponentially during S-phase [43] (Fig 5D, gray line). Because MS analyses are routinely normalized to the overall amount of protein in each sample, this increase in protein mass per cell is not reflected in steady-state protein measurements. Therefore, accumulation of proteins synthesized predominantly during S-phase may be masked by the overall increase in protein mass (Fig 5D, red line).

Still, not all gene products involved in DNA replication share the same pattern. Others show a simultaneous increase in mRNA and translation levels during G1, leading to detectable protein accumulation by S-phase (Fig 5E). These include DNA replication and damage response factors e.g. licensing factor MCM2, Claspin (CLSPN), Denticleless (DTL) and PCNA, as well as FAM111B whose function is unknown but may be related to DNA replication, as predicted by its pattern of expression. Taken together, these observations suggest that the specific time a protein is synthesized may determine whether or not it would appear to oscillate at the steady-state proteome level. Furthermore, the different expression patterns of histones at the mRNA, translation and protein levels are a good example for the important information that can be gained by combining multi-omic measurements.

### Expression dynamics of cytoplasmic versus mitochondrial translation machineries

In addition to histones, many other gene products showed an increase in protein abundance upon transition to S-phase. Among those, we identified a group of genes involved in mitochondrial translation. To determine if this is a general pattern for the mitochondrial translation machinery, we followed the oscillations of all genes encoding for mitochondrial ribosomal proteins and tRNA synthetases in our unfiltered Z-transformed dataset. Indeed, we detected an S-phase specific increase in protein abundance for these genes, which was associated with mild increases in translation at G1 (S9A and S9B Fig), suggestive of delayed accumulation kinetics. In contrast, genes encoding for cytoplasmic ribosomal proteins and tRNA synthetases showed a different pattern; their relative abundance peaked during mitosis and decreased during S-phase (S9C and S9D Fig).

### Utility of multi-omic comparisons for detecting pathway- and gene-specific cell cycle periodicity

**Fig 6:**
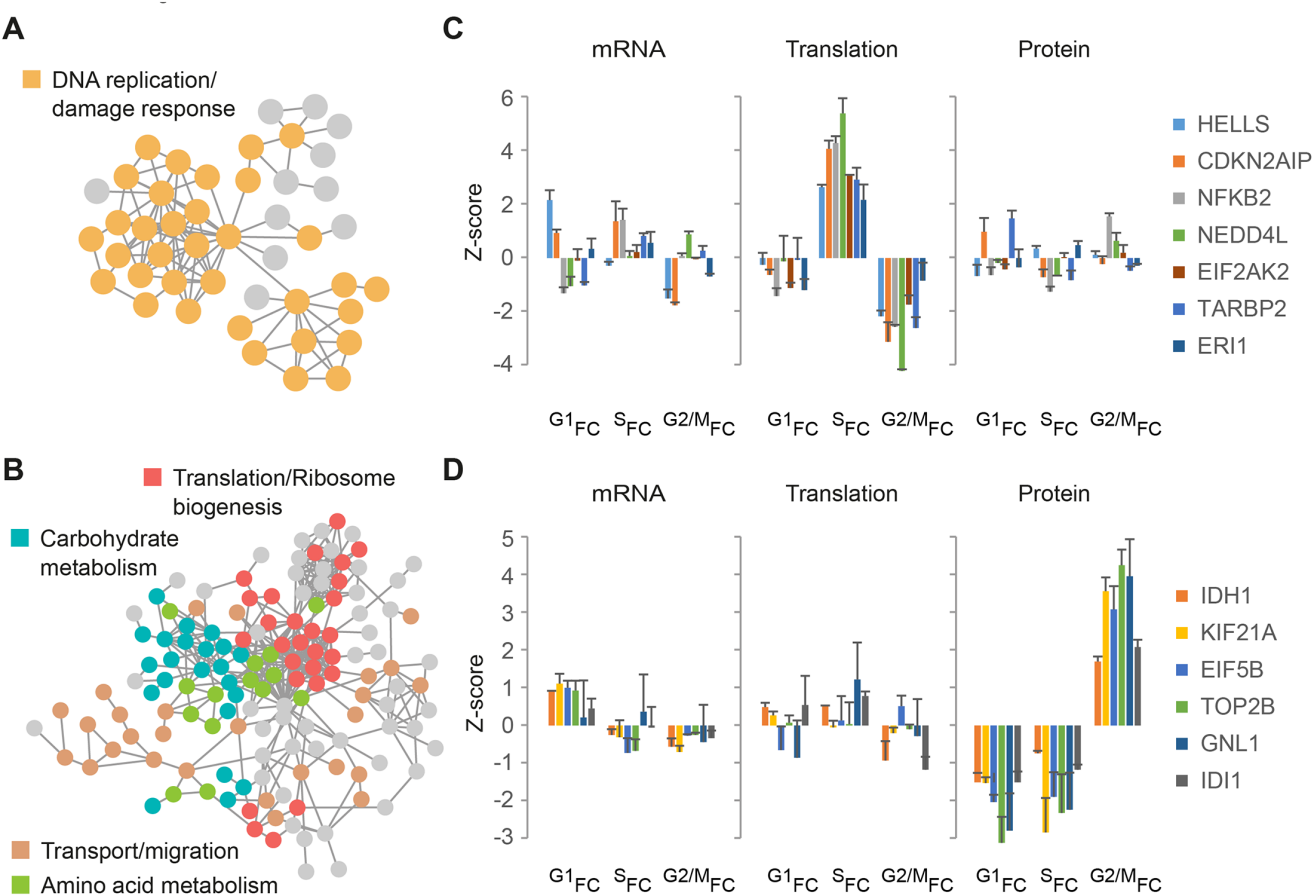
Network analysis. **(A-B)** STRING network analysis of gene products from Fig 4 clusters C (**A**) and E (**B**), with STRING interaction confidence > 0.5. Selected functional groups are indicated in different colors. **(C-D)** Bar plots show mRNA, translation and protein fold-change ratios for selected gene products from cluster C and E. G1_FC_, S_FC_ and G2/M_FC_ represent fold-change ratios relative to the previous cell cycle phase i.e. G2/M-to-G1, G1-to-S, and S-to-G2/M, respectively. Error bars represent +SD of triplicate measurements.

Combining datasets that measure different levels of gene expression under similar experimental conditions can lend further support to the discovery of novel gene products with potential cell cycle functions. For example, a similar pattern of change at two or more levels of expression can allow higher confidence in assigning cell cycle periodicity to specific gene products, even if the change in expression is mild. Furthermore, gene products that share a common pattern of expression with known cell cycle regulators may function in the same pathways. To probe our dataset for functional interactions, we used the STRING database and generated 2 separate protein association maps for gene products from clusters C and E (Fig 4), which are dominated by increased S-phase translation and M-phase protein accumulation, respectively. While the vast majority of gene products in cluster C have an annotated role in DNA replication or damage response (Fig 6A and S10 Fig), gene products in cluster E are involved in a variety of processes e.g. translation and ribosome biogenesis, carbohydrate and amino acid metabolism, transport and migration (Fig 6B and S10 Fig). These include gene products not previously shown to have periodic expression or cell cycle specific functions. For example, translation of the ubiquitin E3 ligase NEDD4L was found to peak during S-phase (Fig 6C, green) while motor protein KIF21A accumulates at the protein level during mitosis (Fig 6D, yellow).

To experimentally validate the potential of our approach to identify novel cycling gene products, we performed immunoblot and qPCR analyses of several candidates found to be predominantly regulated at the steady-state protein level during mitosis. For this purpose, we synchronized HeLa cells by double thymidine block and harvested the synchronized populations at 2, 4, 6, 8, 10 and 12 hours after release from the second block. Antibodies and oligonucleotides specific for Pyruvate kinase (PKM), D-3-phosphoglycerate dehydrogenase (PHGDH), Isocitrate dehydrogenase (IDH1) and DNA topoisomerase 2-beta (TOP2B) were used to measure protein and mRNA, in parallel to the cell cycle markers CCNA2, CCNB1 and AURKA. This analysis showed that, as revealed by the proteomic data, protein amounts of these genes peak during G2/M (S11A Fig) while their mRNA levels remain constant throughout the cell cycle (S11B Fig). This oscillatory pattern may suggest a previously unknown role for these proteins in cell cycle progression. These results suggest a novel link between cell cycle progression and metabolism that may be necessary to provide the energy and the necessary building blocks for cell growth.

## Discussion

### Integrative analysis of the cell cycle transcriptome, translatome and proteome

In this work, we present the first integrative analysis of the intricate relationship between mRNA, translation and protein levels along the mammalian cell cycle. Consistent with previous reports [9,10], our data show that both mRNA and translation levels explain a large proportion of the variation in protein abundance (R^2^ = 0.23 and 0.45, respectively), supporting a substantial role for translation regulation in modulating steady-state protein amounts throughout the cell cycle. This is in contrast with a recent work that reported a much higher contribution of mRNA levels to both absolute protein abundance as well as changes in protein levels upon LPS stimulation of dendritic cells [8]. However, even if our analyses underestimate the contribution of mRNA levels due to methodological errors or noise, as previously suggested [44], the imperfect correlations between translation and protein levels indicate that post-translational regulation of protein abundance contributes significantly to the diversity of the proteome.

To minimize potential biases due to inter-platform variance, we analyzed the fold-change differences between each pair of consecutive cell cycle phases. While this eliminates much of the variance, differences in dynamic range between platforms may still affect the outcome of such analyses. The extent of the true dynamic range of mRNA and protein in biological systems is still largely controversial; for example, RNA-seq exhibits a wider dynamic range as compared to microarrays, but this depends on the analytical depth or number of reads [36,45]. Similarly, the dynamic range of the proteome depends on technical parameters e.g. measurement time and analytical depth. Direct comparisons of RNA-seq and deep proteomic analysis have shown that for the same genes, the spread of the proteome is wider than that of the corresponding mRNA [38]. However, true ranges can only be determined by absolute quantification of proteins and mRNAs. In the current work, to avoid underestimation of transcriptional control due to the lower dynamic range of microarrays, we used Z-score transformation. While this approach is a commonly-accepted standard in gene expression studies, it may also lead to overcorrection and thus underestimation of the impact of translation or protein degradation. To address this possibility, we confirmed our main conclusions using the raw untransformed dataset.

Analysis of Z-transformed differences revealed that translation levels tend to change concordantly with mRNA levels, particularly as cells progress through mitosis, indicating that the mitotic gene expression program is largely dominated by changes at the mRNA level. This conclusion is supported by a previous study showing that translational control plays a less pervasive role during mitosis as compared to G1- and S-phases [18]. However, this normalization may have led to underestimation of the effect of inhibition of translation elongation during mitosis, which we have previously shown [24,26]. Even after normalization, not all cycling gene products are regulated at the mRNA level; expression of specific subsets of genes appears to fluctuate entirely at the level of translation and/or protein degradation. Furthermore, in both the Z-transformed and untransformed datasets, we observed that a large proportion of changes in mRNA or translation levels are not reflected in the steady-state proteome. This may be explained by delayed protein accumulation, which is further confounded by dilution effects related to cell growth, as exemplified by histone expression dynamics (Fig 5). Another possible explanation for this disagreement is rapid degradation of newly-synthesized proteins. Due to the rapid life cycle of mRNA and the experimental design of PUNCH-P, both the transcriptome and the translatome represent a snapshot of gene expression, while proteome measurements reflect the accumulation of proteins with time. Therefore, stable proteins may be seen to accumulate without increased synthesis, while labile proteins may be highly synthesized but still escape detection at the steady-state proteome level. For example, the p53 tumor suppressor gene, which is known to be transcribed, translated and rapidly degraded in HeLa cells [46,47], was indeed detected only at the mRNA and translation levels. Additionally, other gene products encoding for labile proteins e.g. Hypoxia-inducible factor 1 (HIF1A) [48], Cyclin E2 (CCNE2) [49] and Securin (PTTG1) [50] completely escape detection in the steady-state proteome, but show periodic expression at the level of mRNA and/or translation. Finally, we cannot rule out the possibility that patterns not observed in the proteome may emerge by sampling a larger number of time points along the cell cycle.

**Fig 7:**
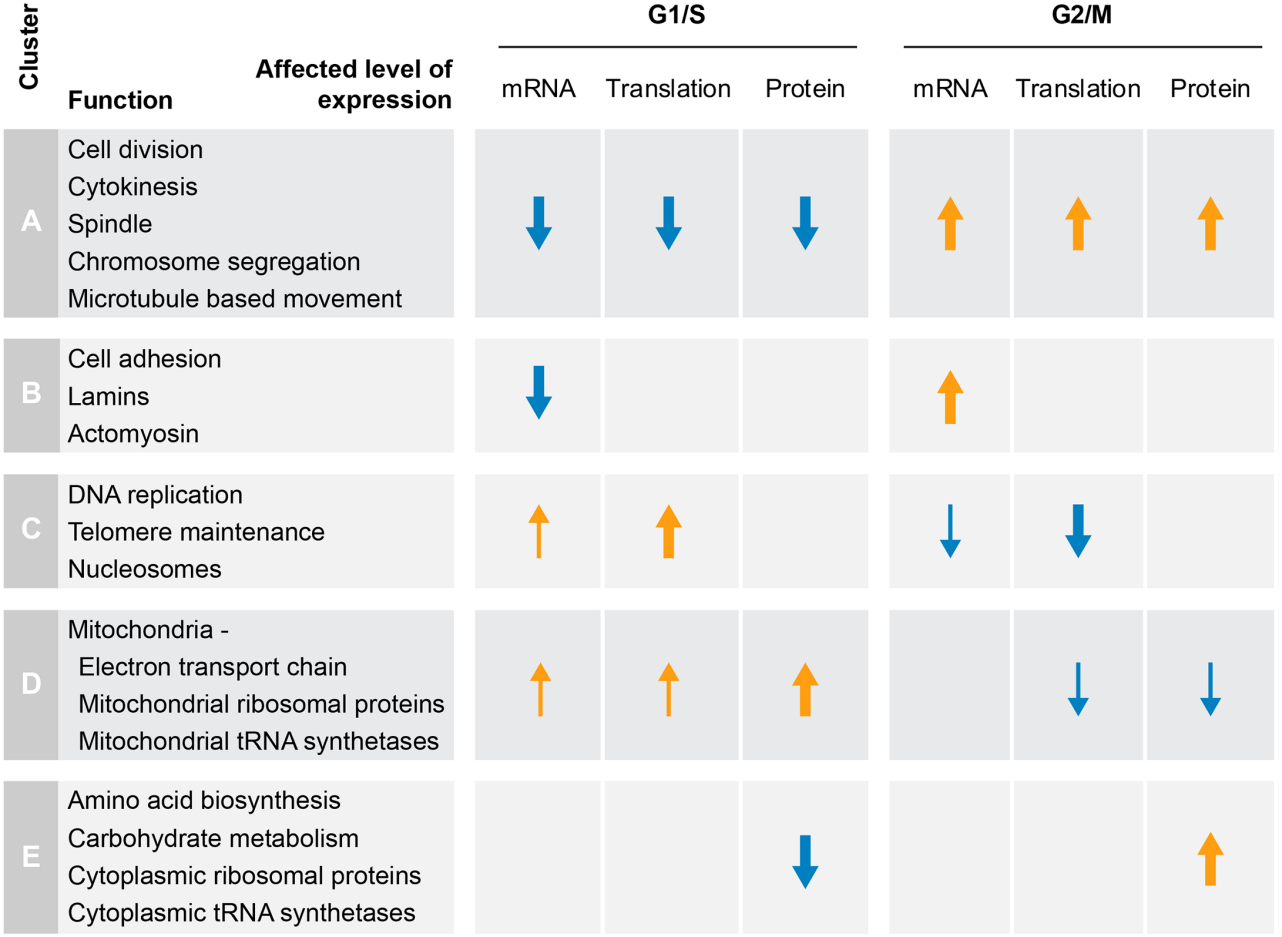
Functional gene expression changes along the cell cycle. Our data suggest several main patterns of gene expression (related to Fig 4): concordant increases in mRNA, translation and protein levels upon entry to mitosis and subsequent reductions upon exit from mitosis, representative of classic mitotic functions (Cluster A); increased mRNA levels during mitosis, which are buffered at the translation and protein levels, encoding for cell adhesion proteins and lamins (Cluster B); increased S-phase translation with little change in mRNA levels or protein abundance, including DNA replication and chromatin assembly factors (Cluster C); increased mRNA and translation levels at G1 with delayed protein accumulation at S-phase, e.g. mitochondrial proteins (Cluster D); and accumulation of protein during mitosis or increased degradation during S-phase with little or no change in mRNA or translation levels, e.g. major biosynthetic pathways and the cytoplasmic translation machinery (Cluster E). Orange and blue arrows indicate increased and decreased expression, respectively. Arrow width represents relative extent of increase or decrease.

Clustering of significantly-changing gene products reveals distinct patterns of expression, as outlined in Fig 7. Concordant increases in mRNA, translation and protein are characteristic of genes with mitotic functions (cluster A), while accumulation of protein without underlying changes in mRNA or translation is characteristic of major biosynthetic pathways e.g. carbohydrate and amino acid metabolism (cluster E). S-phase, on the other hand, is dominated by two main patterns of gene expression; delayed accumulation of proteins that are synthesized throughout G1, e.g. mitochondrial genes (cluster D), and increased translation of proteins involved in DNA replication (cluster C), which is buffered by the increase in cell size and therefore remains undetectable at the steady-state protein level. Another unusual buffering effect is observed for genes with increased mRNA levels during mitosis (cluster B), which is associated with a minor effect on translation and no apparent effect on protein levels. This cluster consists of e.g. lamins and cell adhesion genes and may either represent non-functional cycling of mRNA or indicate that these changes in expression can be observed at the translation and protein levels only by increasing the temporal resolution of sampling.

### Regulation at the level of protein stability and expression dynamics of cytoplasmic versus mitochondrial translation machineries

While most genes with known mitotic functions are regulated at the mRNA level, with consequent effects on translation levels and protein abundance, there are hundreds of other gene products that peak during mitosis but are mostly controlled by differential protein stability. This pattern is characteristic of major metabolic pathways e.g. carbohydrate and amino acid biosynthesis (Fig 6B), whose protein products are reduced at S-phase or elevated upon entry to mitosis without detectable changes in mRNA or translation levels. A similar pattern was also detected for the cytoplasmic translation machinery, including ribosomal proteins and tRNA synthetases (S9 Fig). It is known that ribosomal proteins are synthesized in excess to ensure that efficient ribogenesis is never limited by the available supply of protein components [51]. This is balanced by simultaneous proteasomal degradation to prevent accumulation of free ribosomal proteins that causes ribosomal stress and cell cycle arrest [52,53]. Therefore, reduced stability at S-phase may represent increased degradation of unincorporated ribosomal proteins to prevent ribosomal stress and allow unhindered progression through the G1/S checkpoint. Because degradation of ribosomal proteins occurs predominantly in the nucleus [51], this process may be inhibited after nuclear envelope breakdown, resulting in re-stabilization of ribosomal proteins during mitosis as suggested by our data. Furthermore, our findings are supported by another study that showed increased protein expression of tRNA synthetases during mitosis in yeast [54]. Interestingly however, this pattern of expression is not shared by the mitochondrial translation machinery. Synthesis of mitochondrial ribosomal proteins and tRNA synthetases peaks at G1, followed by steady-state accumulation of protein at S-phase. This increase in mitochondrial components, which presumably reflects proliferation of mitochondria prior to doubling of the cytoplasmic proteome, is consistent with the high energetic demands associated with DNA replication and cell division, as well as the need to guarantee redistribution of sufficient number of mitochondria between the daughter cells. Remarkably, ribosomal and mitochondrial proteins were recently found to be the two main groups regulated at the level of protein synthesis and degradation in LPS-stimulated dendritic cells [8].

### Regulation at the level of translation

As described above, we observed surprising oscillations in expression of cytoplasmic translation machinery components, including periodic expression of mRNA binding proteins and molecular chaperones. It is tempting to speculate that such oscillations represent adaptations of the protein synthesis machinery for optimal translation of specific mRNAs. In agreement with this concept, it has been shown that cell cycle-regulated genes in both yeast and human cells have different codon preferences, with a bias towards non-optimal codons [54,55]. Here we found a subset of gene products whose translation levels increased during S-phase with little to no change in mRNA levels (Cluster C). These include DNA replication and damage response factors (FANCI, XRCC1, PARP2, DNA polymerases, RFC proteins) as well as kinases and transcription modulators (CDK2, NFKB2, KLF5). Interestingly, synthesis of DNA damage response proteins in yeast was shown to involve codon adaptation and tRNA modifications [56]. It is possible that similar mechanisms are at work in mammals, conferring S-phase specific translation regulation as reflected in our data.

### Combining multi-omic expression data as a means of exploring functional significance

Similarity in expression patterns often reflect shared functions. Therefore, combining methods that represent distinct steps of gene expression may lend greater power to detecting unique patterns and thus assigning related functions. This is true for gene products that show either concordant or discordant changes at the different levels of expression but share similar patterns with genes of known function. One specific example is FAM111B, an under-characterized gene associated with a human skin and lung disorder [57], whose expression pattern is remarkably similar to that of genes with functions in DNA replication (Fig 5E). Based on this expression signature, we propose that FAM111B should be studied for a possible role in DNA replication or another S-phase specific function. Interestingly, its paralog FAM111A was recently identified to be involved in DNA replication [58], but shows a distinct cycling pattern in our data (Cluster C, S4 Table).

In summary, our work provides information on gene products and pathways that were not previously shown to have periodic expression, and it is tempting to speculate that these play yet unknown roles in progression or regulation of the mammalian cell cycle. Taken together with the observation that some patterns are observed only at the level of mRNA, translation or protein, this highlights the importance and utility of combining expression data from different platforms and measurement techniques, to generate unique insights into the complex nature of gene expression regulation.

## Materials and Methods

### Cell synchronization

HeLa S3 cells were grown in DMEM (Invitrogen) supplemented with 10% fetal calf serum, 2 mM L-glutamine, and 100 U/mL penicillin/streptomycin (Biological Industries). For double-thymidine block, cells were treated with 2 mM thymidine for 19 h, released from G1/S block in fresh DMEM for 9 h, treated again with 2 mM thymidine for 18 h, released in fresh DMEM, and harvested at different time points, as indicated.

### Western blotting

Whole cell extracts were separated by 8% SDS-PAGE, transferred to polyvinylidene difluoride (PVDF) membrane and probed with anti–AURKA (C4718T, 1:1000, Cell Signaling Technology); anti-CCNA2 (H432, 1:1000, Santa Cruz Biotechnology); anti-CCNB1 (ab72, 1:1500, Abcam); anti-IDH1 (8137S, 1:1000, Cell Signaling Technology); anti-PHGDH (13428S,1:1000, Cell Signaling Technology); anti-PKM (3190S, 1:1000, Cell Signaling Technology); anti-TOP2B (HPA024120, 1:250, Prestige antibodies, Sigma); anti-TUB (T6074,1:400000, Sigma) at 4°C, overnight. 12.5% gels were used similarly and probed with anti-Total H3 (ab1791, 1:5000, Abcam); anti-phosphoH3 (ab5176, 1:1000, Abcam). Secondary antibodies, donkey-anti-mouse (715-035-151, 1:10000, Jackson ImmunoResearch Laboratories) or anti-rabbit (711-035-152, 1:10000, Jackson ImmunoResearch Laboratories) were conjugated to horseradish peroxidase antibodies and incubated for 1 h at room temperature. Blots were visualized with the Pierce ECL Western blotting substrate (34087, Thermo Scientific) according to manufacturer’s instructions.

### Real time quantitative-PCR

Total RNA was isolated from cell pellets with TRIzol (15566026, Life Technologies) and 1 μg of RNA was subjected to reverse transcriptase-PCR with random hexamers using the Verso cDNA synthesis kit (AB–1453/A, Thermo scientific). Real time quantitative-PCR (RTqPCR) was performed using the PerfeCTa SYBR Green FastMix, ROX (95073–12, Quanta Biosciences). The following primers synthesized from Integrated DNA Technologies were used and expression levels were normalized to GAPDH expression. GAPDH Fwd (5’-GCACCGTCAAGGCTGAGAAC–3’), GAPDH Rev (5’-ATGGTGGTGAAGACGCCAGT–3’); HIST1H3 Fwd (5’-AGGACTTTAAGACCGACCT–3’), HIST1H3 Rev (5’-ATGATGGTGACCCGTTTG–3’); AURKA Fwd (5’-AGGACCTGTTAAGGCTACA–3’), AURKA Rev (5’-GAGCCTGGCCACTATTTAC–3’); CCNA2 Fwd (5’-TAGATGCTGACCCATACCTC–3’), CCNA2 Rev (5’-GATTCAGGCCAGCTTTGTC–3’); CCNB1 Fwd (5’-GCACCAAATCAGACAGATGG–3’), CCNB1 Rev (5’-CGACATCAACCTCTCCAATC–3’); UNG Fwd (5’-TCTCCCTTGCCTTTATGGTG–3’), UNG Rev (5’-CACCCCAACATCTGTCACTG–3’); PCNA Fwd (5’-GTGAACCTCACCAGTATGTC–3’), PCNA Rev (5’-CCAAGGTATCCGCGTTATC–3’); PKM Fwd (5’-CATTCATCCGCAAGGCATC–3’), PKM Rev (5’-TCATCAAACCTCCGAACCC–3’); PHGDH Fwd (5’-TTGCTGTTCAGTTCGTGGAC–3’), PHGDH Rev (5’-GAGCTTCTGCCAGACCAATC–3’); IDH1 Fwd (5’-ACAGGAGACGTCCACCAATC–3’), IDH1 Rev (5’-TTGCAAAGAAGGCAAGCTCT–3’); TOP2B Fwd (5’-GGTACTGGATGGGCTTGTA–3’), TOP2B Rev (5’-GTTTGGAAGCATGGGATGAG–3’).

### Sample preparation and MS analysis

Cell pellets were lysed in Urea buffer containing 6 M urea/2 M thiourea in 100 mM Tris-HCl (pH 8.5) at room temperature. Protein concentrations were determined using the Bradford Protein assay (Bio-Rad). Equal amounts of protein (10 μg) from each sample lysate were reduced with 1 mM dithiothreitol (DTT) and alkylated with 5 mM Iodoacetamide (IAA). Protein digestion was performed for three hours with endoprotease LysC (Wako chemicals; 1:100 enzyme to protein ratio) followed by an overnight digestion with sequencing grade modified Trypsin (Promega; 1:50 enzyme to protein ratio) at room temperature. Peptides were acidified with TFA and purified on C18 stageTips [59]. Eluted peptides were separated using EASY-nLC–1000 HPLC system (Thermo Scientific) coupled to the Q Exactive Plus MS (Thermo Scientific) through an EASY-spray ionization source. Peptides were separated on a 50 cm long PepMap EASY-spray column (Thermo Scientific) using four-hour gradients of water:acetonitrile (with 0.1% formic acid). MS analysis was performed in a data dependent mode using a top 10 method for MS/MS acquisition. Analysis was performed with the Maxquant Software [60] (version 1.5.2.10) and MS/MS spectra were searched against the UniprotKB database (Nov2014) with the Andromeda search engine [61]. The label free quantification algorithm was used for data normalization [62] with minimum ratio count set to 2. FDR was set to 1% at both the peptide-spectrum match and protein levels. FDR was determined by the target-decoy approach.

The mass spectrometry proteomics data have been deposited to the ProteomeXchange Consortium [63] via the PRIDE partner repository with the dataset identifier PXD002802.

### Data analysis

Microarray dataset GSE26922 (Affymetrix Human Gene 1.0 ST Array) was downloaded from Gene Expression Omnibus (GEO), with RMA-normalized expression values for timepoints 2, 8 and 12h. Translation and protein measurements (log2) were from previous work (timepoints 2, 8.5 and 14h) [2] and this study (timepoints 2, 8.5 and 14h), respectively. To align the three datasets, an ID conversion list was generated using BioMart with the following attributes: (a) Affymetrix Microarray HuGene 1.0 st v1 probeset ID(s) and (b) UniProt/SwissProt Accession, based on Homo sapiens genes (GRCh37.p13). The three datasets were combined using Perseus into a protein-centric matrix, with each row representing a protein group. This was done as follows: first, translation levels and protein abundance measurements were combined based on Uniprot IDs to generate a union of all proteins detected in at least one of the two dataset; then mRNAs levels were added using the maximum value option to select only the most abundant transcripts coding for indistinguishable protein groups. For comparison of absolute abundance, the iBAQ algorithm [9] was used to normalize translation and protein levels. Alternative absolute quantification based on the Top three proteins was calculated from the MaxQuant peptide table, using R. For fold-change comparisons, translation and protein levels were normalized using the LFQ algorithm with a minimum of 2 ratio counts, to achieve higher quantitative accuracy. Means were calculated from a minimum of 2 replicates. Fold-change values for mRNA, translation and protein were calculated by subtracting the mean logarithmized value from the preceding cell cycle phase. GOBP cell cycle filtering consisted of the following categories: Cell cycle, Cell cycle arrest, Cell cycle checkpoint, Cell cycle cytokinesis, Cell cycle phase, Cell cycle process and Cell division. For histone gene expression, due to multiplicity of genes coding for the same protein products, only transcripts showing highest fold-change ratios were selected for further analysis.

### Bioinformatic analysis

Statistical analysis was done with Perseus using one-sample Student’s t-test (FDR<0.05), to extract the z-transformed values that significantly differ from zero in each sample. Data are presented as means ± SD, where indicated. Spearman’s correlations were determined separately for each replicate and averaged. Corrected Spearman’s correlations were calculated according to Csardi et al. [40]. Generic k-means clustering (Fig 4, S7 Fig) was performed on Z-transformed logarithmized fold-change ratios. Hierarchical clustering for S8 Fig was performed on untransformed logarithmized fold-change ratios using correlation distances and with column tree order preserved. Enrichment analysis was performed using Fisher’s exact test with an FDR value of 0.02. Protein networks were constructed in the STRING database with interaction confidence > 0.5 and visualized using Cytoscape.

## Supporting Information

S1 Fig Analysis of cell cycle synchronization using double-thymidine block. HeLa S3 cells were synchronized using double-thymidine block and harvested at 2, 8.5 and 14 hours after release from the second block, corresponding to S, G2/M and G1 phases. Samples were subjected to flow cytometry analysis of DNA content using propidium iodide.
(TIF)

S2 Fig Association of translation and steady-state protein levels with protein stability. Scatterplot of protein versus translation levels for G1 phase, colored according to protein half-life as determined using published pSILAC measurements. Green and red represent highly labile and highly stable proteins, respectively. As expected, proteins previously reported to have short half-lives are mostly found below the trend line, while those with long half-lives are mostly found above the trend line.
(TIF)

S3 Fig qPCR validation of cell cycle markers. HeLa cells were synchronized using double-thymidine block and harvested at 2, 4, 6, 8, 10, 12 and 14 hours after release from the second block. RNA was extracted and subjected to qPCR analysis using primers specific to the indicated transcripts. Relative expression values are normalized to GAPDH level and shown as bar graphs (gray), with error bars representing +SD of triplicate measurements. Corresponding microarray values from Sadasivam et al. are shown as line plots (green).
(TIF)

S4 Fig Effect of absolute protein quantitation method on correlations. Unsupervised hierarchical clustering of Spearman’s rank correlation of RMA-normalized mRNA levels versus iBAQ- or TOP3-normalized translation and protein levels.
(TIF)

S5 Fig Corrected Spearman’s rank correlations, related to Fig 2. Spearman’s rank correlations before (green) and after (purple) correction as described by Csardi et al. 2015 to control for technical variability. Error bars represent +SD of triplicate measurements.
(TIF)

S6 Fig Expression of the same gene products increases in mitosis and decreases in G1. Scatterplots of fold-change ratios of mRNA (**A**), translation (**B**), and protein (**C**) for S-to-G2/M_FC_ versus G2/M-to-G1_FC_. Gene products with GOBP cell cycle annotations are highlighted purple.
(TIF)

S7 Fig Clustering of periodic gene products, related to Fig 4. K-means clustering of gene products showing statistically-significant changes (one-sample T-test of Z-transformed fold-changes, FDR<0.05) along the cell cycle in at least one of mRNA, translation and/or protein levels. Each panel represents a distinct cluster with a separate heatmap **(A)** and profile plot **(B)** reporting Z-transformed values for fold-change mRNA, translation and protein levels. G1, S and G2/M represent fold-change ratios relative to the previous cell cycle phase i.e. G2/M-to-G1, G1-to-S, and S-to-G2/M, respectively. (**C**) Fisher enrichment scores for the clusters F-J (FDR <0.02, selected categories). The complete enrichment analysis is included in S5 Table.
(TIF)

S8 Fig Hierarchical clustering of non-Z scored fold-change ratios, related to Fig 4. Unsupervised hierarchical clustering of gene products showing changes of >1.5 fold-change along the cell cycle in at least one of mRNA, translation and/or protein levels. Heatmap shows the complete unedited clustering results of fold-change ratios (**A**), while profile plots show corresponding Z-score clusters from Fig 4 (**B**). G1, S and G2/M represent fold-change ratios relative to the previous cell cycle phase i.e. G2/M-to-G1, G1-to-S, and S-to-G2/M, respectively.
(TIF)

S9 Fig Pattern of change for cytoplasmic and mitochondrial components of the translation machinery. Boxplots of fold-change mRNA, translation and protein levels for the following categories: (**A**) Mitochondrial 28S and 39S ribosomal proteins; (**B**) Mitochondrial tRNA synthetases; (**C**) Cytoplasmic 40S and 60S ribosomal proteins; (**D**) Cytoplasmic tRNA synthetases. G1_FC_, S_FC_ and G2/M_FC_ represent fold-change ratios relative to the previous cell cycle phase i.e. G2/M-to-G1, G1-to-S, and S-to-G2/M, respectively.
(TIF)

S10 Fig STRING network analysis, related to Fig 6. STRING network analysis of gene products from Fig 4 clusters C and E, with STRING interaction confidence > 0.5. Selected functional groups are indicated in different colors.
(TIF)

S11 Fig Validation of novel cycling proteins. HeLa cells were synchronized by double-thymidine block and harvested at 2, 4, 6, 8, 10 and 12 hours after release from the second block. Protein and mRNA were extracted and subjected to immunoblot (**A**) and qPCR analysis (**B**) using antibodies and primers specific to the indicated genes as described in the methods section.
(TIF)

S1 Table Combined dataset of log(2) RMA-normalized mRNA levels, LFQ- and iBAQ-normalized translation rates, and LFQ- and iBAQ-normalized protein abundance, for G1, S-phase and G2/M. (XLSX)

S2 Table 1D Enrichment of functional annotations (FDR<0.02) based on protein stability score, calculated as the ratio of steady-state abundance to translation rate for each protein. Low and high scores represent functions enriched for labile and stable proteins, respectively.
(XLSX)

S3 Table Gene products whose levels increase (Z-score > 2). (XLSX)

S4 Table Gene products with statistically significant changes along the cell cycle, in at least one level of expression, Z-transformed (one-sample t-test, FDR<0.05). (XLSX)

S5 Table Fisher functional enrichment of Clusters A-J. (XLSX)

S6 Table Cyclic gene products with a cutoff of > 1.5 fold change, across the cell cycle, in at least one level of expression, raw non-transformed values. (XLSX)
