# Supplementary figures and images for: Uncovering Hidden Layers of Cell Cycle Regulation through Integrative Multi-omic Analysis
Source: PLoS Genet. 2015 Oct 6;11(10):e1005554. doi: 10.1371/journal.pgen.1005554 (PMC4595013; doi:10.1371/journal.pgen.1005554)

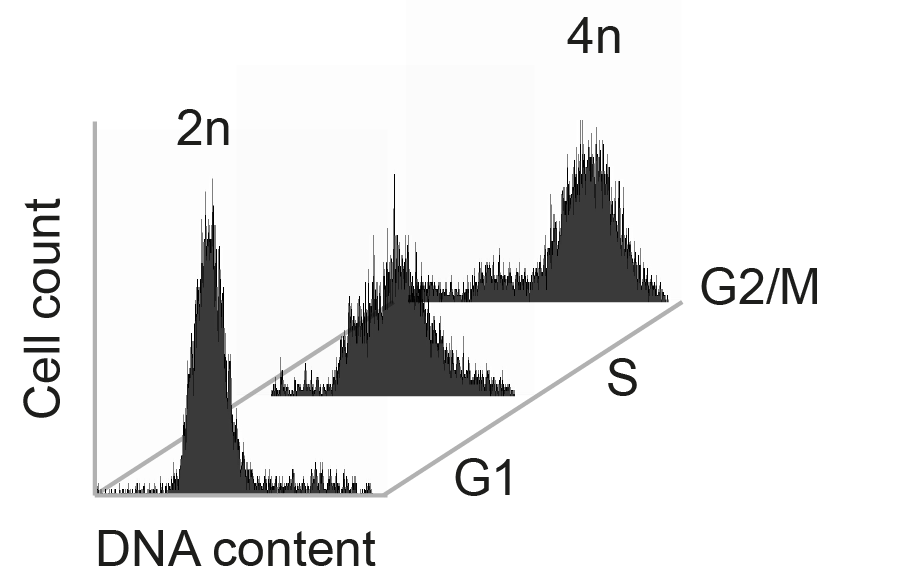

Supplement: S1 Fig — HeLa S3 cells were synchronized using double-thymidine block and harvested at 2, 8.5 and 14 hours after release from the second block, corresponding to S, G2/M and G1 phases. Samples were subjected to flow cytometry analysis of DNA content using propidium iodide. (TIF) [file pgen.1005554.s001.tif]

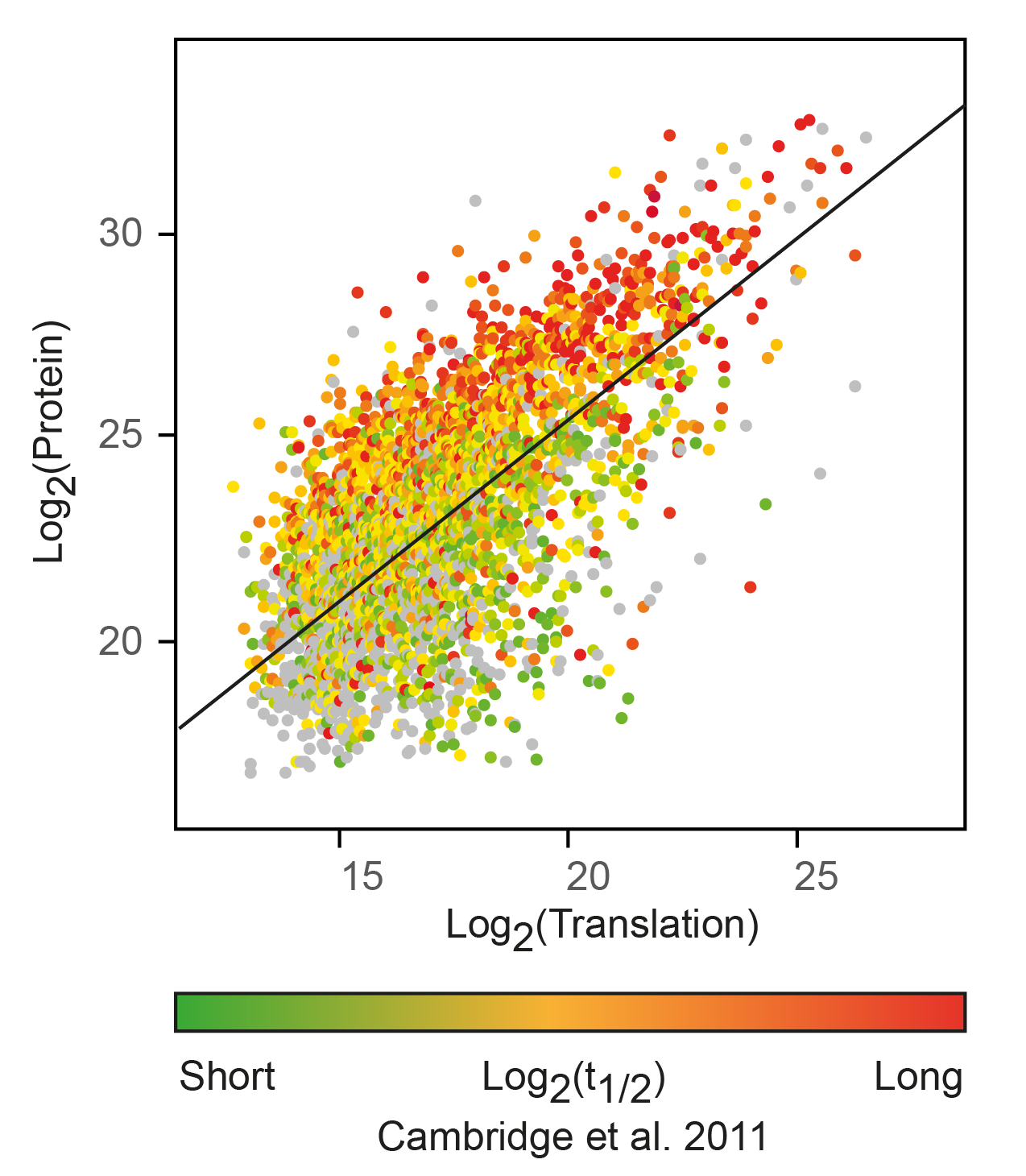

Supplement: S2 Fig — Scatterplot of protein versus translation levels for G1 phase, colored according to protein half-life as determined using published pSILAC measurements. Green and red represent highly labile and highly stable proteins, respectively. As expected, proteins previously reported to have short half-lives are mostly found below the trend line, while those with long half-lives are mostly found above the trend line. (TIF) [file pgen.1005554.s002.tif]

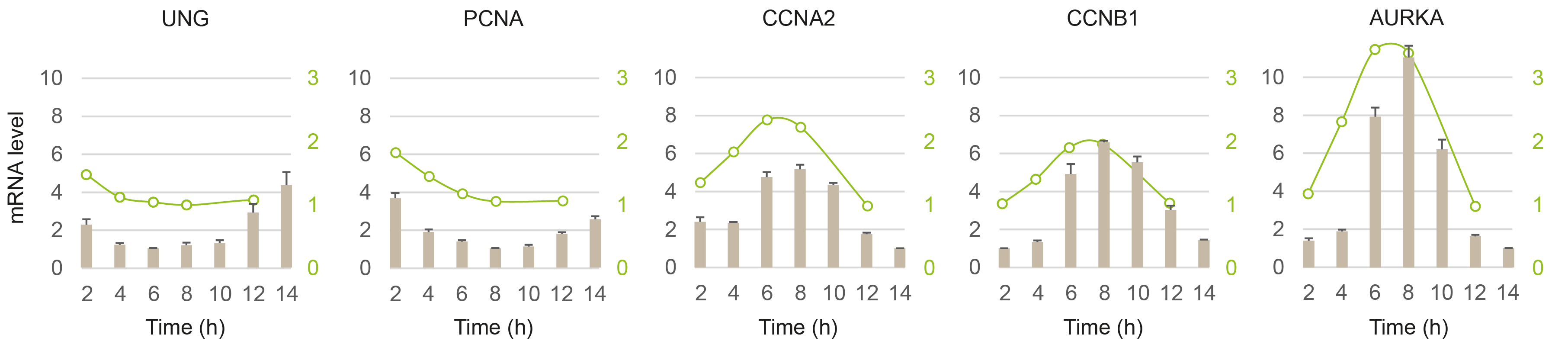

Supplement: S3 Fig — HeLa cells were synchronized using double-thymidine block and harvested at 2, 4, 6, 8, 10, 12 and 14 hours after release from the second block. RNA was extracted and subjected to qPCR analysis using primers specific to the indicated transcripts. Relative expression values are normalized to GAPDH level and shown as bar graphs (gray), with error bars representing +SD of triplicate measurements. Corresponding microarray values from Sadasivam et al. are shown as line plots (green). (TIF) [file pgen.1005554.s003.tif]

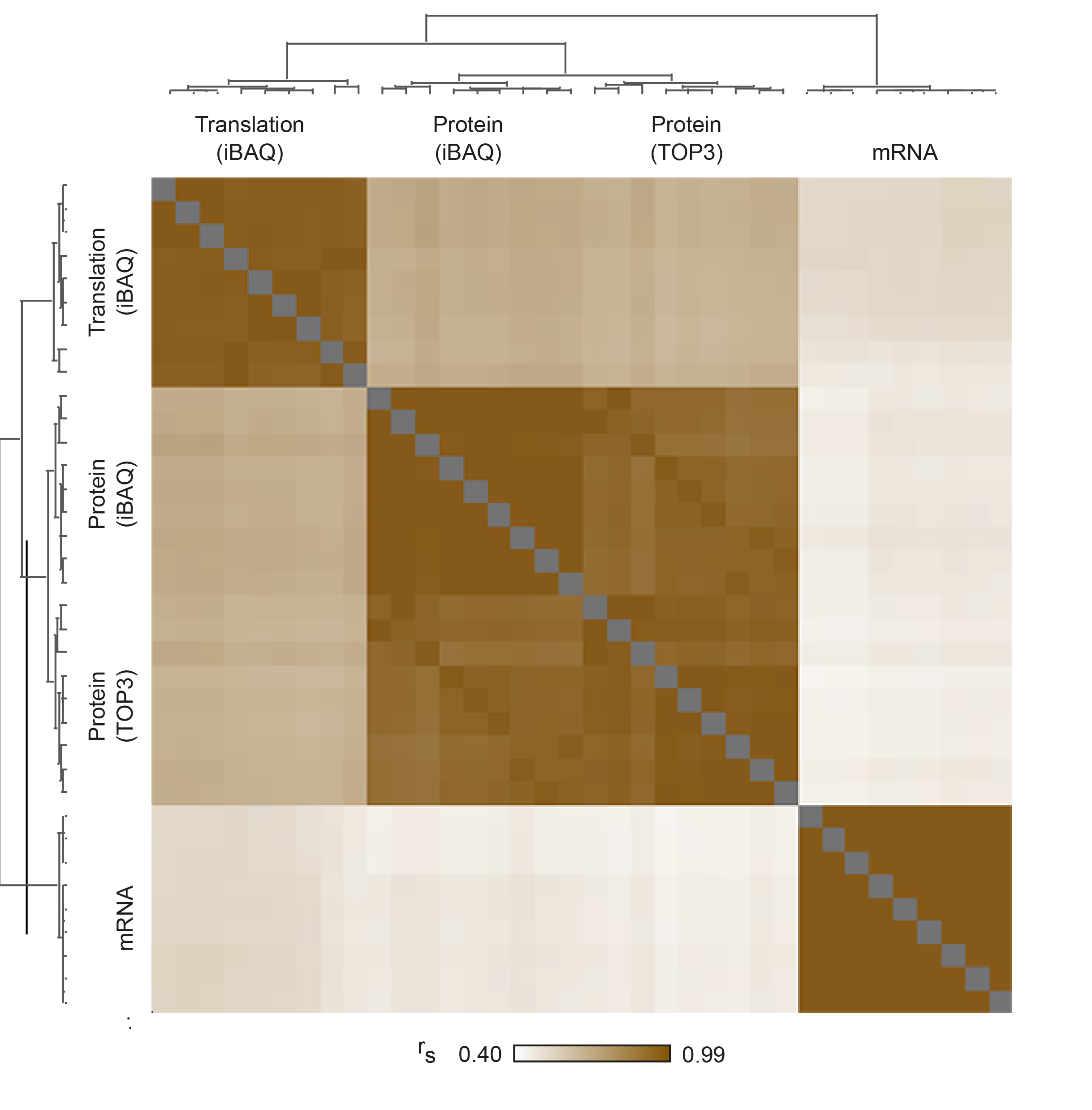

Supplement: S4 Fig — Unsupervised hierarchical clustering of Spearman’s rank correlation of RMA-normalized mRNA levels versus iBAQ- or TOP3-normalized translation and protein levels. (TIF) [file pgen.1005554.s004.tif]

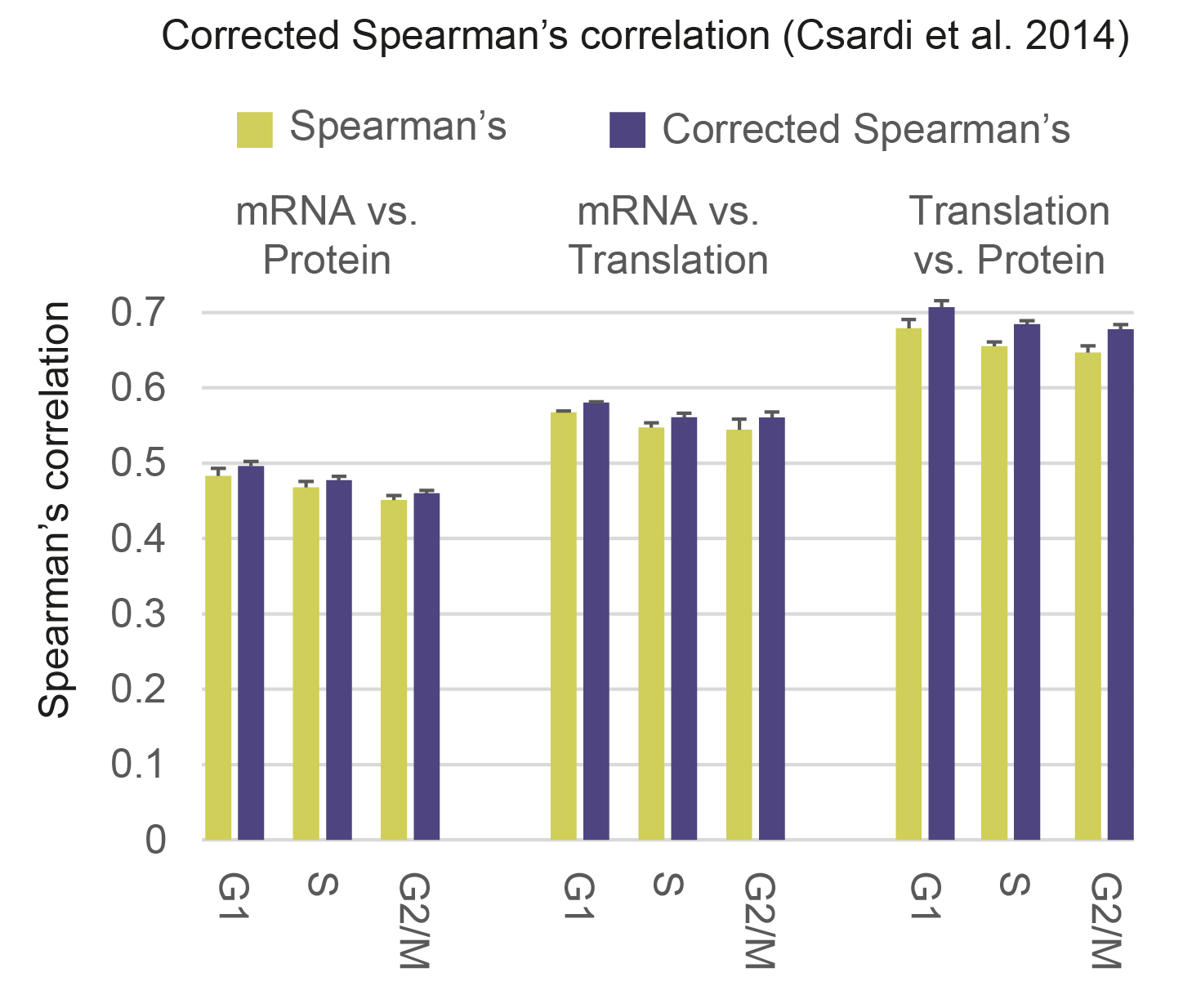

Supplement: S5 Fig — Spearman’s rank correlations before (green) and after (purple) correction as described by Csardi et al. 2015 to control for technical variability. Error bars represent +SD of triplicate measurements. (TIF) [file pgen.1005554.s005.tif]

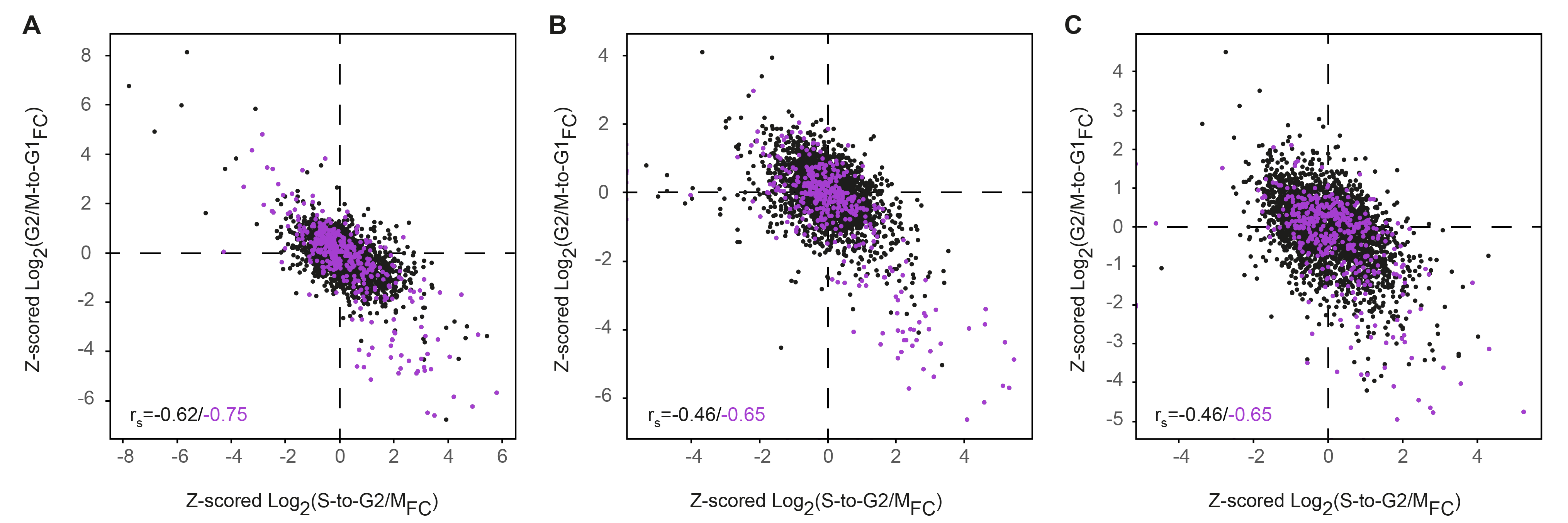

Supplement: S6 Fig — Scatterplots of fold-change ratios of mRNA (A), translation (B), and protein (C) for S-to-G2/MFC versus G2/M-to-G1FC. Gene products with GOBP cell cycle annotations are highlighted purple. (TIF) [file pgen.1005554.s006.tif]

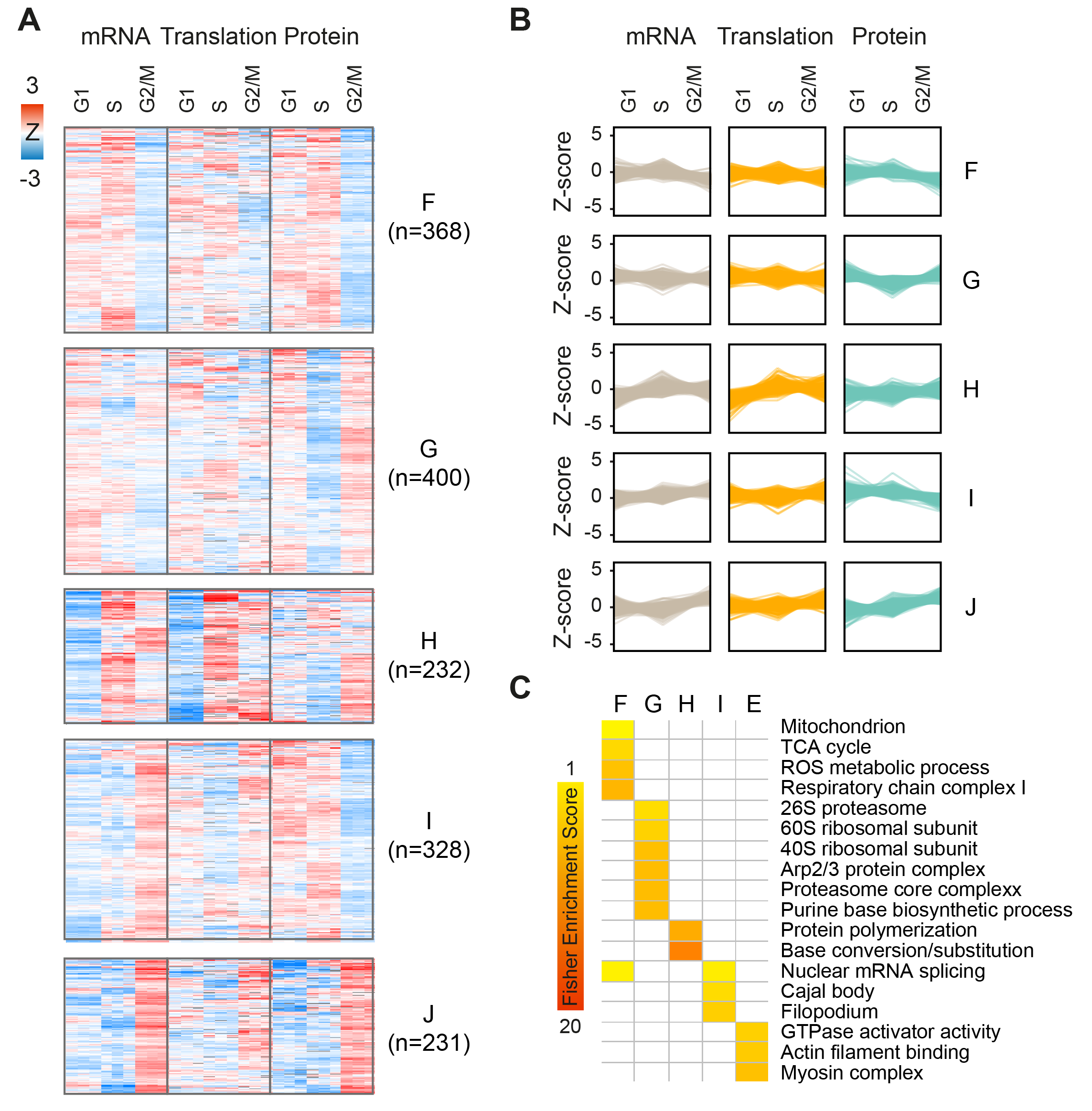

Supplement: S7 Fig — K-means clustering of gene products showing statistically-significant changes (one-sample T-test of Z-transformed fold-changes, FDR<0.05) along the cell cycle in at least one of mRNA, translation and/or protein levels. Each panel represents a distinct cluster with a separate heatmap (A) and profile plot (B) reporting Z-transformed values for fold-change mRNA, translation and protein levels. G1, S and G2/M represent fold-change ratios relative to the previous cell cycle phase i.e. G2/M-to-G1, G1-to-S, and S-to-G2/M, respectively. (C) Fisher enrichment scores for the clusters F-J (FDR <0.02, selected categories). The complete enrichment analysis is included in S5 Table. (TIF) [file pgen.1005554.s007.tif]

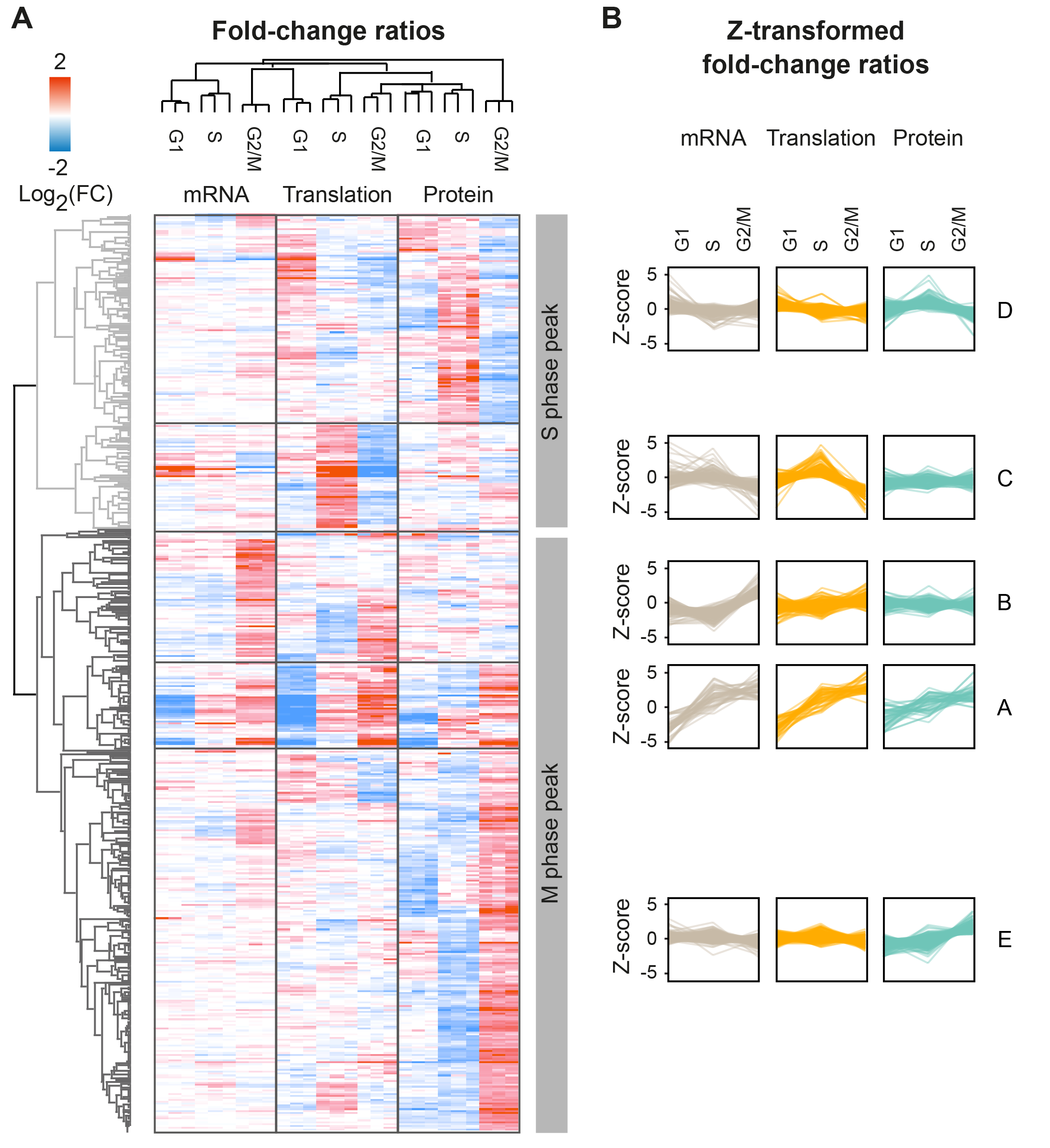

Supplement: S8 Fig — Unsupervised hierarchical clustering of gene products showing changes of >1.5 fold-change along the cell cycle in at least one of mRNA, translation and/or protein levels. Heatmap shows the complete unedited clustering results of fold-change ratios (A), while profile plots show corresponding Z-score clusters from Fig 4 (B). G1, S and G2/M represent fold-change ratios relative to the previous cell cycle phase i.e. G2/M-to-G1, G1-to-S, and S-to-G2/M, respectively. (TIF) [file pgen.1005554.s008.tif]

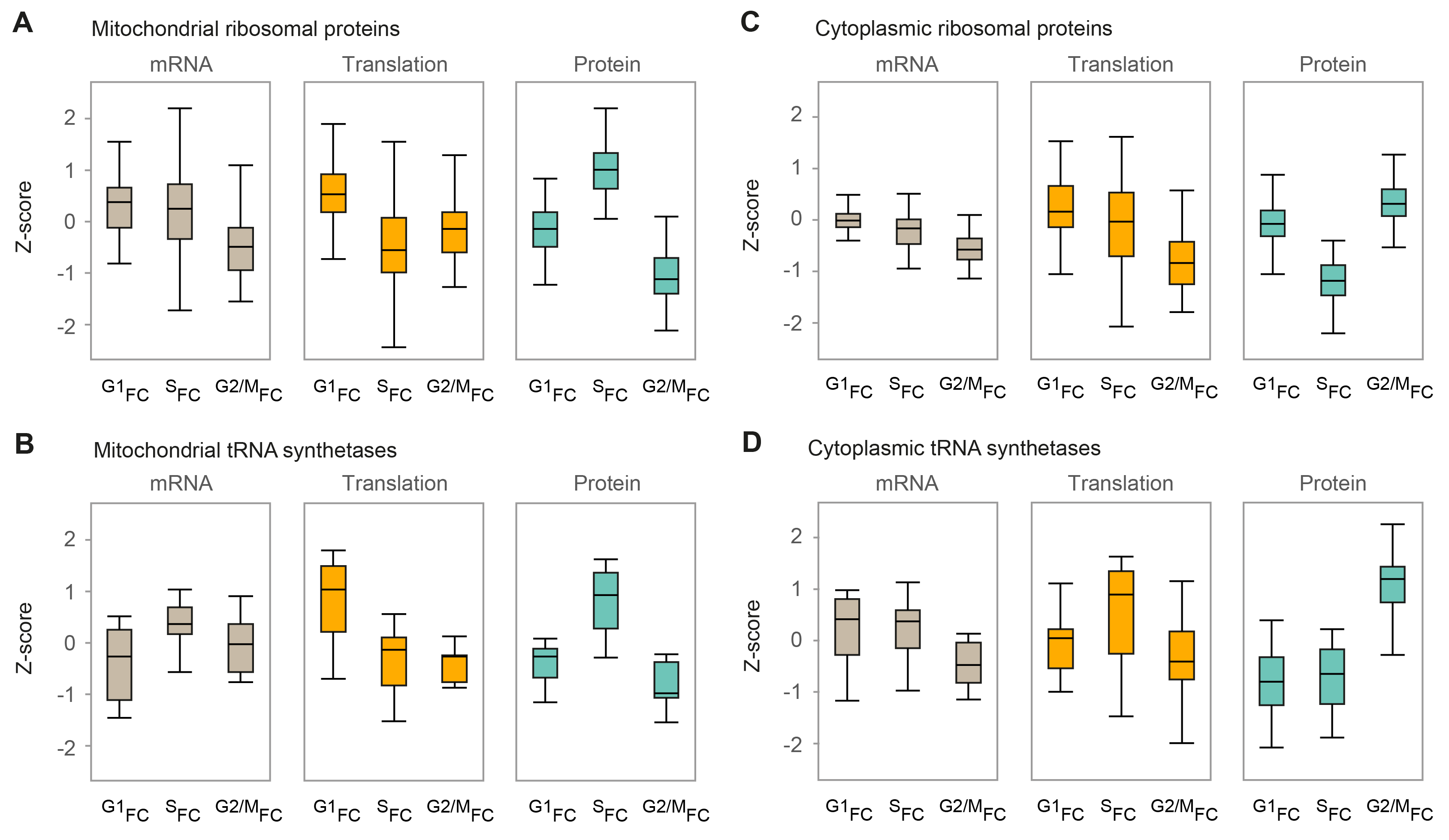

Supplement: S9 Fig — Boxplots of fold-change mRNA, translation and protein levels for the following categories: (A) Mitochondrial 28S and 39S ribosomal proteins; (B) Mitochondrial tRNA synthetases; (C) Cytoplasmic 40S and 60S ribosomal proteins; (D) Cytoplasmic tRNA synthetases. G1FC, SFC and G2/MFC represent fold-change ratios relative to the previous cell cycle phase i.e. G2/M-to-G1, G1-to-S, and S-to-G2/M, respectively. (TIF) [file pgen.1005554.s009.tif]

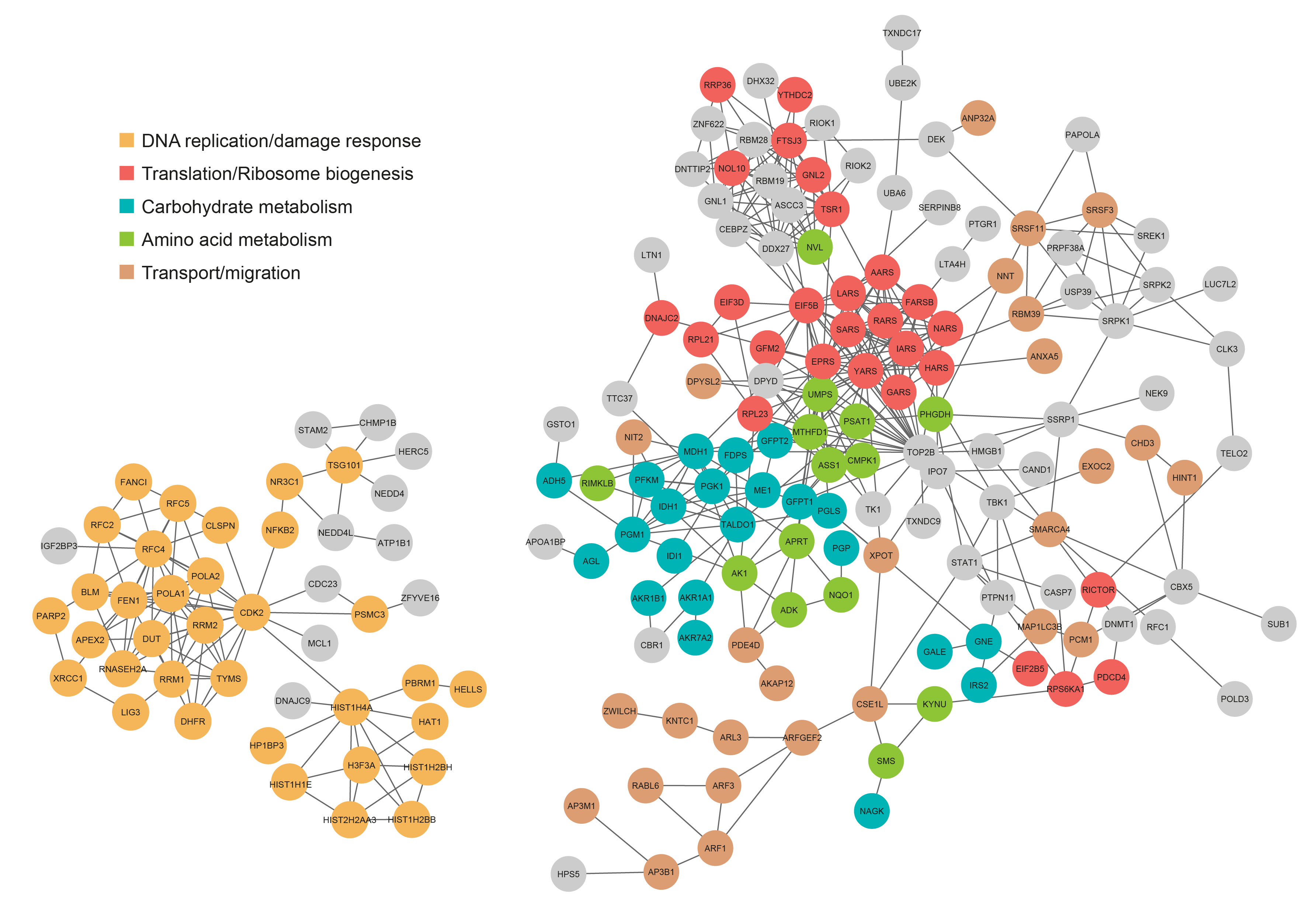

Supplement: S10 Fig — STRING network analysis of gene products from Fig 4 clusters C and E, with STRING interaction confidence > 0.5. Selected functional groups are indicated in different colors. (TIF) [file pgen.1005554.s010.tif]

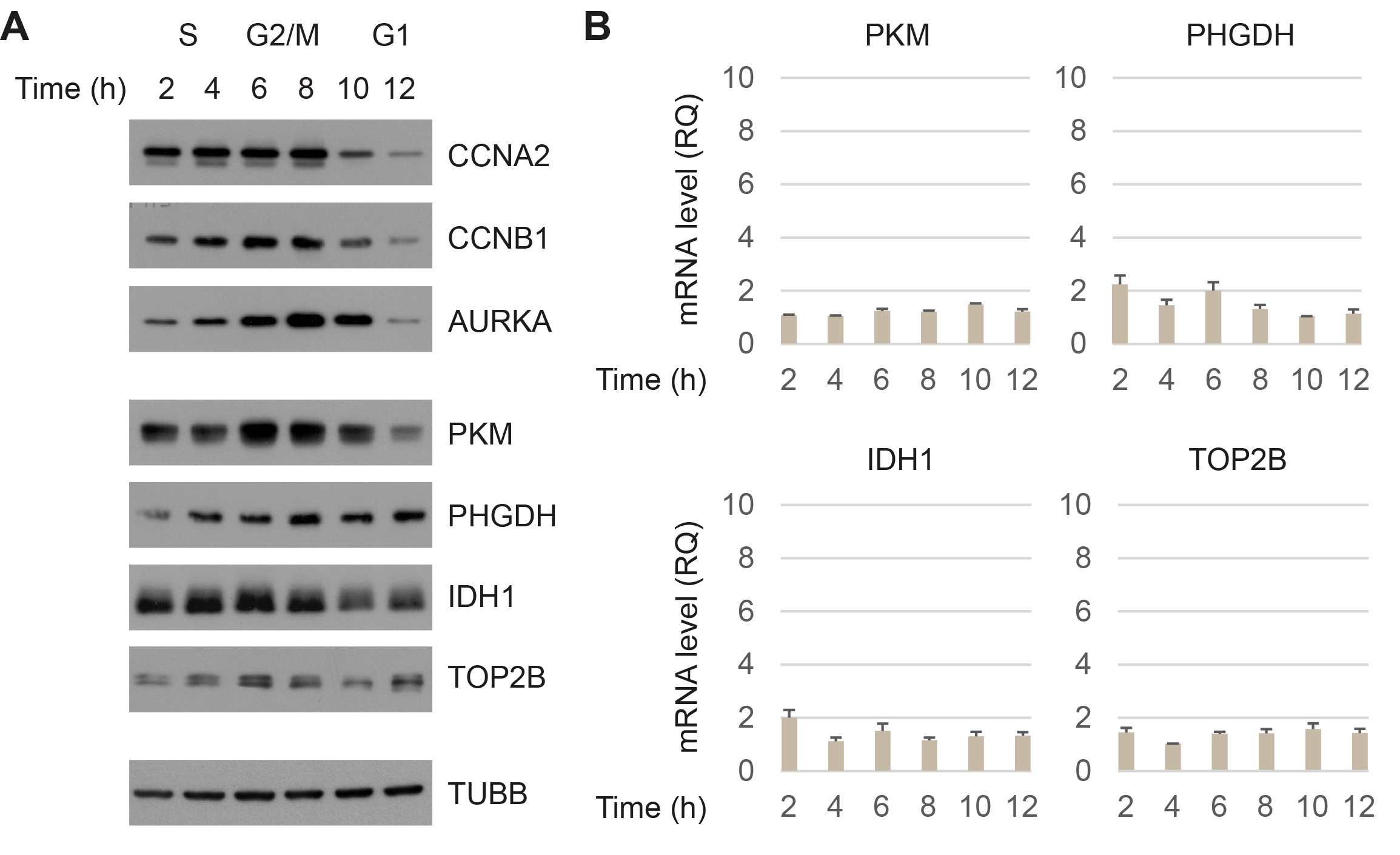

Supplement: S11 Fig — HeLa cells were synchronized by double-thymidine block and harvested at 2, 4, 6, 8, 10 and 12 hours after release from the second block. Protein and mRNA were extracted and subjected to immunoblot (A) and qPCR analysis (B) using antibodies and primers specific to the indicated genes as described in the methods section. (TIF) [file pgen.1005554.s011.tif]
